# Global burden of cardiovascular diseases attributable to diet low in fruits from 1990 to 2021 and forecasting the future trends: A population-based study

**DOI:** 10.1097/MD.0000000000044189

**Published:** 2026-01-16

**Authors:** Yuanxiu Hong, Yuzhe Kong, Yu Zuo

**Affiliations:** a Central South University Hospital, Changsha, China; b Xiangya School of Medicine, Central South University, Changsha, China.

**Keywords:** cardiovascular diseases, disease burden, epidemiology, fruit, mortality forecasting

## Abstract

This study explores the worldwide burden of cardiovascular diseases (CVD) associated with insufficient fruit consumption, utilizing insights from the 2021 Global Burden of Disease Study. The analysis focused on the global, regional, and national consequences of low fruit intake on CVD. It investigated variations across different demographics, including age and gender, and explored the relationship between disease burden and the socio-demographic index. Additionally, an ARIMA model was employed to forecast CVD trends linked to dietary habits through 2050. The 2021 data revealed that inadequate fruit consumption contributed to roughly 1.41 million deaths and 35.32 million disability-adjusted life years from CVD, with a declining pattern observed over recent years. The elderly population, especially those aged 75 and above, and males were particularly susceptible. Future projections suggest a likely increase in CVD incidents in low-socio-demographic index regions, with African countries potentially experiencing heightened challenges by 2030 and 2050 due to poor fruit intake. The study highlights the critical need for preventive strategies aimed at reducing CVD by improving dietary behaviors, specifically by increasing fruit consumption.

## 1. Introduction

Globally, cardiovascular diseases (CVD) stand as the predominant cause of death and illness, affecting 523 million individuals and resulting in 18.6 million deaths in 2019 alone.^[1–6]^ These conditions exert a significant socioeconomic and medical strain, especially in countries with lower economic resources.^[7]^ Given this context, dietary modifications, particularly increasing fruit and vegetable intake, are increasingly emphasized as key preventative measures.^[8–11]^

Public health guidelines across various nations advocate for increased fruit and vegetable consumption to combat chronic conditions such as CVD and diabetes. A comprehensive meta-analysis involving over 4 million participants from recent cohort studies indicated that those with the highest intake of fruits and vegetables had a reduced risk of total CVD by 7%, coronary heart disease by 12%, and stroke by 18%, compared to those with the lowest intake.^[12]^ This trend held true across various groups including adolescents,^[13]^ women,^[14]^ individuals with diabetes,^[15]^ and persistent smokers.^[16]^ The promotion of fruit and vegetable consumption as a standalone strategy to reduce CVD risk is increasingly recognized.^[17]^ However, much of this research has been limited to Western countries, leaving the relationship between fruit and vegetable consumption and CVD risk unclear and inconsistent globally.^[15,18–21]^

Thus, to narrow the current research gap, this investigation utilizes 2021 data on global disease burdens to assess the impact of diets deficient in fruits on CVD and employs an ARIMA model to predict future patterns, supported by prior research.^[22,23]^

## 2. Methods

### 2.1. Study population

Utilizing data from the 2021 global burden of disease study, this research reviewed 369 diseases and injuries, as well as 87 risk factors, in 204 countries spanning from 1990 to 2021.^[24–28]^

To evaluate the influence of cardiovascular diseases (CVD), we applied methodologies from prior research.^[29]^ We refined the raw data sourced from various methods including health surveys and verbal autopsies to enhance accuracy.^[30]^ This refined data underwent analysis using the cause of death ensemble model, yielding yearly, area-specific estimates of CVD mortality by age and sex.^[29,31]^ A comparative risk assessment identified key risk factors for CVD, and population attributable fractions were calculated to estimate the impact of insufficient fruit intake on CVD rates. These population attributable fractionss helped estimate the mortality and disability-adjusted life years (DALYs) related to CVD across different populations and periods.^[24]^ DALYs combined data on years lost due to premature death and years lived with disability, adjusted for severity.^[32]^ The Socio-Demographic Index (SDI) was also developed using indicators such as fertility rates in women under 25 (TFU25), education attainment over age 15 (EDU15+), and income per capita, classifying 204 regions into 5 SDI categories.^[32–34]^

### 2.2. Ethic approval

Our research involved secondary analyses using publicly available databases, thus negating the need for further ethical approvals.

### 2.3. Statical analysis

Age-adjusted rates (AAR) were used to standardize mortality and DALY figures for diverse demographic and age groups. We analyzed these rates using linear regression on their natural logarithms, formatted as y = α + βx + ε, with ‘x’ representing the year and ‘y’ the natural log of the rate. The estimated annual percentage change (EAPC) was determined by the formula 100 × (e^β − 1), accompanied by a 95% confidence interval (95% CI). An upward trend in AAR was identified if both the EAPC and the lower 95% CI limit were positive, a decline if both the EAPC and the upper 95% CI limit were negative, and a stable pattern if neither condition was met.^[35,36]^ We explored the relationship between AAR and SDI using Gaussian process regression with Loess smoothing, and conducted Spearman rank correlation tests to assess this association.^[34,36,37]^

Additionally, an ARIMA model was used to forecast the impact of low fruit intake on future CVD trends globally, regionally, and nationally from 2020 to 2050. In the ARIMA model, “p” refers to the number of lag observations, “q” to the lag of forecast errors, and “d” to the required differencing for data stabilization, with model selection based on the Akaike Information Criterion and Bayesian Information Criterion.^[38]^

Statistical analysis was conducted via R 4.3.2. *P* <.05 was considered as statistically significant.^[39–41]^

## 3. Result

### 3.1. Spatiotemporal patterns of CVD attributable to diet low in fruits

In 2021, low fruit consumption was linked to approximately 1.41 million deaths and 35.32 million DALYs from cardiovascular diseases (CVDs). The age-standardized mortality rate (ASMR) was 16.8024 (95% UI, 7.3635–24.8582), and the age-standardized DALY rate (ASDR) was 411.9148 (95% UI, 155.3696–613.7015) per 100,000 people. Over the past 30 years, there has been a marked global reduction in the impact of CVDs related to low fruit intake (Table 1, Table S1, Supplemental Digital Content, https://links.lww.com/MD/P934).

**Table 1:** Global and regional deaths and DALYs of CVD attributable to diet low in fruits in 1990 and 2021 in 27 global regions.

Location	Deaths Number in 1990	Deaths Number in 2021	ASMR in 2021	DALY Number in 1990	DALY Number in 2021	ASDR in 2021
Global	1009518.0903 (1533483.3942, 377765.2562)	1408811.5018 (2094303.6908, 605678.7687)	16.8024 (24.8582, 7.3635)	27511622.3891 (42009061.3850, 9256310.8599)	35318448.8420 (52740014.3038, 13184716.6310)	411.9148 (613.7015, 155.3696)
Region						
East Asia	277993.2572 (411697.4193, 123821.1393)	287786.0953 (448259.4728, 129598.5347)	15.3136 (23.6225, 7.1636)	7356002.0581 (10980939.8003, 2822700.9127)	6052713.5855 (9612400.6197, 2367149.6087)	299.3632 (471.2901, 119.9219)
Southeast Asia	86150.5285 (131300.8668, 29803.2043)	128478.3544 (195780.2888, 47478.9516)	20.7060 (31.2843, 8.1768)	2661956.2294 (4062520.6664, 837780.1953)	3710103.2521 (5737367.4217, 1247143.4195)	530.9985 (811.8584, 187.0071)
Oceania	1281.3506 (1979.3156, 447.2331)	2529.9649 (3940.0349, 940.5984)	34.6023 (52.9498, 13.7654)	42146.2360 (65225.8015, 13650.5297)	82627.9390 (129241.2701, 28379.7787)	914.7081 (1420.5203, 340.8124)
Central Asia	17027.4347 (27247.2666, 5216.8751)	16708.8458 (25582.6555, 6084.5422)	23.6117 (36.1483, 8.9542)	451455.2795 (709992.8453, 129734.4971)	419134.0259 (648914.2145, 146159.6333)	508.7162 (788.2766, 181.5692)
Central Europe	38317.4881 (60039.3784, 13350.6056)	35336.4903 (50232.5463, 19661.6938)	15.3239 (21.8077, 8.3362)	918955.4291 (1445512.1327, 290797.5794)	646175.2856 (928158.1238, 333277.2902)	305.8699 (438.6766, 153.0026)
Eastern Europe	88395.4950 (147517.2193, 21700.2650)	79337.5710 (131436.0285, 23670.7796)	22.8153 (37.7888, 6.8039)	2174897.9942 (3546912.6291, 536836.9834)	1712483.2808 (2821109.1990, 493751.2588)	518.7649 (853.4577, 146.7856)
High-income Asia Pacific	20292.8756 (31753.2779, 7576.2155)	22632.3175 (33416.0400, 11568.6672)	3.9751 (6.0542, 1.7150)	488194.4409 (775053.5276, 143247.2354)	399499.3205 (611571.4776, 161354.9829)	99.6285 (155.6203, 31.7009)
Australasia	2819.5900 (4790.5190, 755.1896)	2172.7579 (3431.8706, 882.7577)	3.6784 (5.8319, 1.4703)	61329.4267 (102437.6442, 15157.7779)	39592.5773 (62712.0236, 14528.0615)	78.4588 (122.8083, 27.0537)
Western Europe	57020.1105 (90557.8637, 20821.2455)	53488.6795 (73371.4653, 31408.4074)	4.5672 (6.4050, 2.6168)	1154396.6206 (1851181.3995, 367036.8311)	809831.2610 (1151910.0490, 435418.2960)	85.1998 (123.5215, 42.0456)
Southern Latin America	7436.0915 (10793.9478, 3552.2945)	5767.6866 (7913.8397, 3799.3698)	6.3849 (8.7851, 4.1291)	178759.3811 (268173.7562, 74016.1428)	109452.8915 (156533.9364, 61379.7720)	127.4575 (182.8951, 68.9163)
High-income North America	43589.1265 (70230.3599, 15498.0133)	53831.8306 (75074.2709, 30209.6384)	8.2318 (11.3775, 4.6416)	986422.3135 (1537976.1915, 344627.4882)	1197426.5714 (1639518.0478, 653456.7756)	210.9592 (286.0763, 116.3729)
Caribbean	4347.5472 (6359.3665, 1970.5564)	6354.0145 (8886.5265, 3608.0453)	11.6848 (16.3491, 6.6298)	112714.4345 (166230.3369, 44785.5082)	154583.3369 (221325.9863, 77385.4291)	290.4025 (415.2870, 144.6250)
Andean Latin America	2645.5293 (3795.5254, 1303.3381)	3535.2882 (5147.0401, 1974.2343)	6.1324 (8.9000, 3.4817)	68407.1574 (102337.3445, 25835.1520)	85726.1474 (130657.7985, 38058.3124)	140.7461 (212.8702, 65.4336)
Central Latin America	9528.4067 (13499.2901, 4830.6225)	17058.1698 (25115.6729, 7909.4845)	7.0409 (10.3892, 3.3418)	247731.8877 (361243.4620, 104738.3150)	409554.2505 (615215.2819, 160503.3999)	161.2428 (240.7556, 64.7559)
Tropical Latin America	14931.5701 (21726.0998, 7062.5499)	15512.6363 (21513.4625, 8545.9548)	6.1916 (8.5209, 3.4913)	443152.5760 (658100.7516, 177038.8599)	384886.2781 (556609.2771, 188387.5251)	148.9414 (215.0905, 74.1023)
North Africa and Middle East	47625.3422 (65000.5854, 24518.8934)	61914.1112 (85776.6194, 36566.6810)	15.5834 (21.0890, 9.7005)	1305592.6308 (1826117.7873, 601325.9947)	1672480.7499 (2408874.2320, 866888.5119)	342.2513 (480.7717, 190.0106)
South Asia	220444.6898 (345928.7582, 70679.5680)	497240.3843 (765216.8930, 162883.2824)	35.7059 (54.7861, 12.6914)	6879383.7332 (10633587.9642, 1991344.9575)	14055557.7164 (21704933.6085, 4162705.6307)	897.4576 (1383.6135, 279.7033)
Central Sub-Saharan Africa	7178.7063 (10588.8776, 3539.5568)	17896.2321 (26059.2817, 8794.0281)	41.6357 (58.7185, 22.0313)	207605.7481 (308839.0744, 95727.4692)	498110.4318 (734550.4403, 238476.8638)	884.2391 (1273.4885, 445.4461)
Eastern Sub-Saharan Africa	30287.6322 (41261.8629, 15909.6412)	44093.6476 (61636.0600, 23411.5385)	31.1140 (42.5317, 18.4132)	862770.6514 (1196593.6815, 414739.7941)	1243766.3437 (1758967.6037, 600104.0654)	684.0084 (956.3541, 361.4347)
Southern Sub-Saharan Africa	7826.3270 (11062.9278, 4050.5292)	17248.7045 (24046.7091, 9838.5073)	34.1119 (46.3224, 20.3638)	228692.1390 (326726.8893, 109359.5396)	467006.6838 (658817.2081, 236226.9579)	773.6122 (1085.0791, 420.7271)
Western Sub-Saharan Africa	24378.9913 (35436.4987, 12385.6542)	39887.7193 (57505.2105, 17762.5736)	22.5903 (32.3537, 10.7868)	681056.0219 (993621.2815, 323000.3167)	1167736.9128 (1685260.0557, 498562.0717)	530.6258 (758.8172, 235.9395)
SDI						
High-middle SDI	233536.8068 (365116.6718, 76362.0225)	241437.4580 (372086.2198, 104349.8439)	12.6749 (19.4392, 5.5588)	5978190.5929 (9410326.7653, 1767389.4856)	5050010.9696 (8011842.3789, 1883742.1965)	266.9430 (422.0443, 99.4175)
High SDI	145624.5586 (231499.1537, 50197.1127)	145601.8092 (207321.5596, 78633.0666)	6.4437 (9.2108, 3.3574)	3280429.7362 (5197303.8739, 1021729.8484)	2886921.7230 (4081913.4315, 1415656.7069)	154.4507 (218.7867, 73.0644)
Low-middle SDI	222359.1383 (338353.4437, 82992.7974)	408027.7256 (616330.8326, 151288.1146)	30.2056 (45.1521, 12.2359)	6728310.3346 (10302529.6419, 2200623.4305)	11466776.2940 (17303641.6178, 3760802.5061)	750.0714 (1134.3753, 260.3440)
Low SDI	84439.7227 (123067.8779, 38283.2945)	151017.5850 (217749.0136, 69206.2459)	33.8141 (47.5172, 16.7067)	2475286.5483 (3657828.8925, 1027724.5195)	4302462.8495 (6285144.0878, 1783490.9297)	783.5642 (1126.7662, 352.0367)
Middle SDI	322494.7941 (476215.7190, 137283.0040)	461559.9162 (693942.4752, 195123.7902)	18.7959 (27.9091, 8.4773)	9022020.0583 (13413991.0160, 3350616.7947)	11585361.6529 (17413017.5905, 4343886.1017)	431.4891 (644.8860, 168.2911)

CVD = cardiovascular diseases, DALYs = disability-adjusted life years, SDI = socio-demographic index.

**Figure 1.**
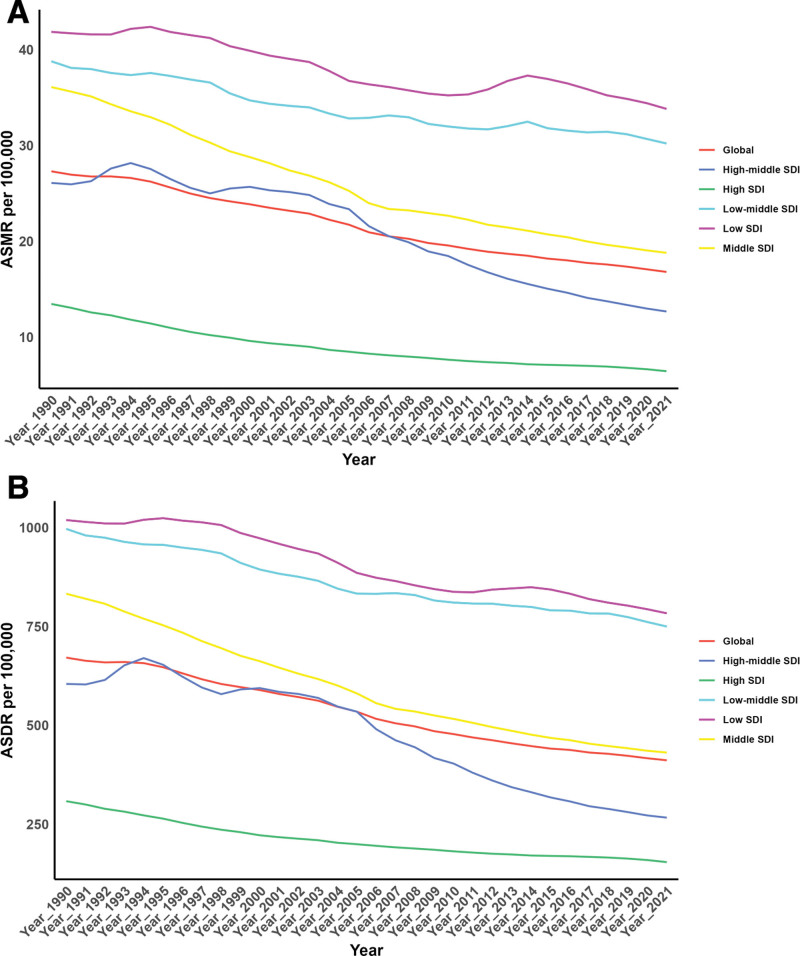
Temporal trends of ASMR and ASDR of CVD Attributable to Diet Low in Fruits from1990 to 2021 in different SDI regions. ASDR = age-standardized DALY rate, ASMR = age-standardized mortality rate, CVD = cardiovascular diseases, DALY = disability-adjusted life year, SDI = socio-demographic index.

Across all SDI regions, significant decreases in ASMR and ASDR due to fruit-deficient diets were observed. (Table 1, Table S1, Supplemental Digital Content, https://links.lww.com/MD/P934, Fig. 1).

Regionally, East Asia and Southeast Asia experienced the highest numbers of CVD deaths and DALYs attributable to low fruit intake. East Asia, South Asia, Central Sub-Saharan Africa, Eastern Sub-Saharan Africa, and Southern Sub-Saharan Africa recorded the highest ASMR and ASDR rates (Table 1).

**Figure 2.**
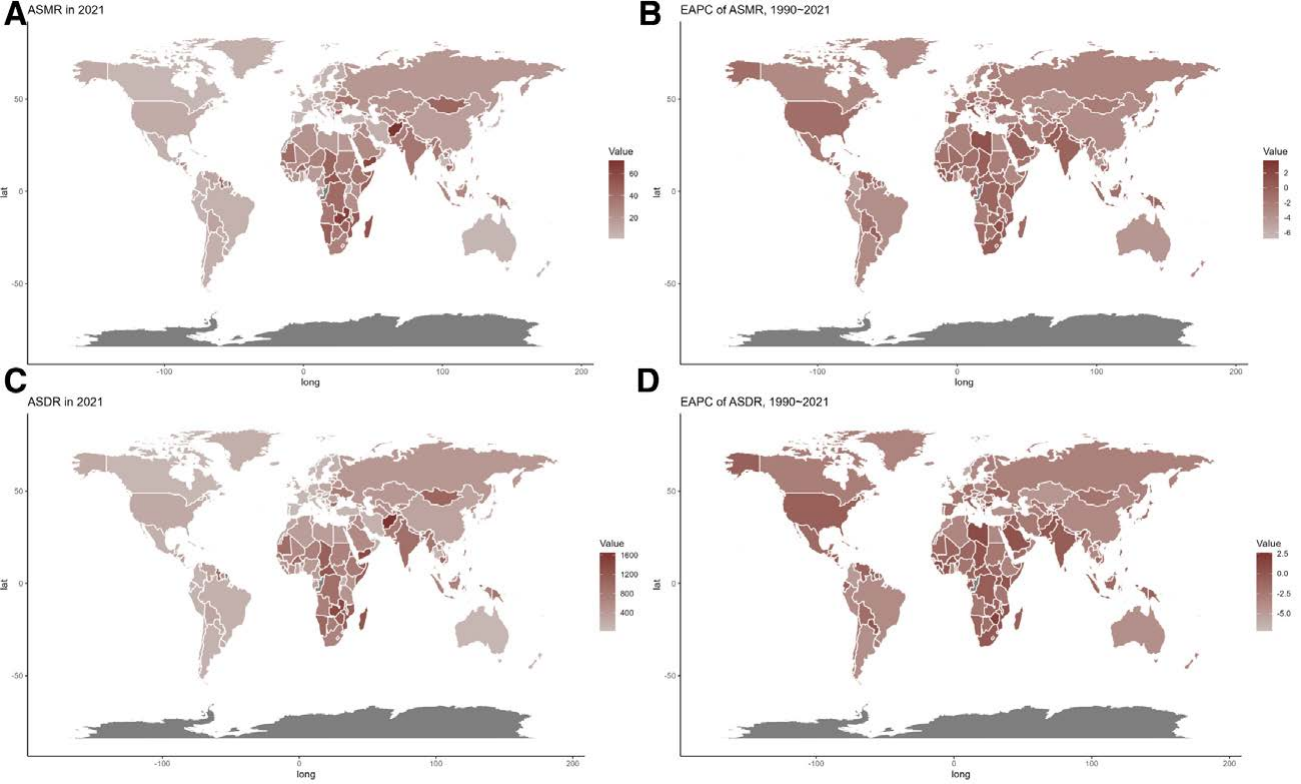
Global distribution of ASMR (A) and ASDR (C) of CVD attributable to diet low in fruits for both sexes in 2021 in 204 countries and territories. EAPC of ASMR (B) and ASDR (D) of CVD attributable to diet low in fruits from 1990 to 2021 in 204 countries and territories. ASDR = age-standardized DALY rate, ASMR = age-standardized mortality rate, CVD = cardiovascular diseases, DALY = disability-adjusted life year, EAPC = estimated annual percentage change.

Nationally, 2021 data revealed considerable variation in ASMR and ASDR related to CVDs from low fruit intake, with countries in Africa and South Asia exhibiting the highest rates. From 1990 to 2021, both ASMR and ASDR showed a general upward trend in many African nations (Table 1, Table S1, Supplemental Digital Content, https://links.lww.com/MD/P934, Fig. 2).

### 3.2. Age and gender pattern

**Figure 3.**
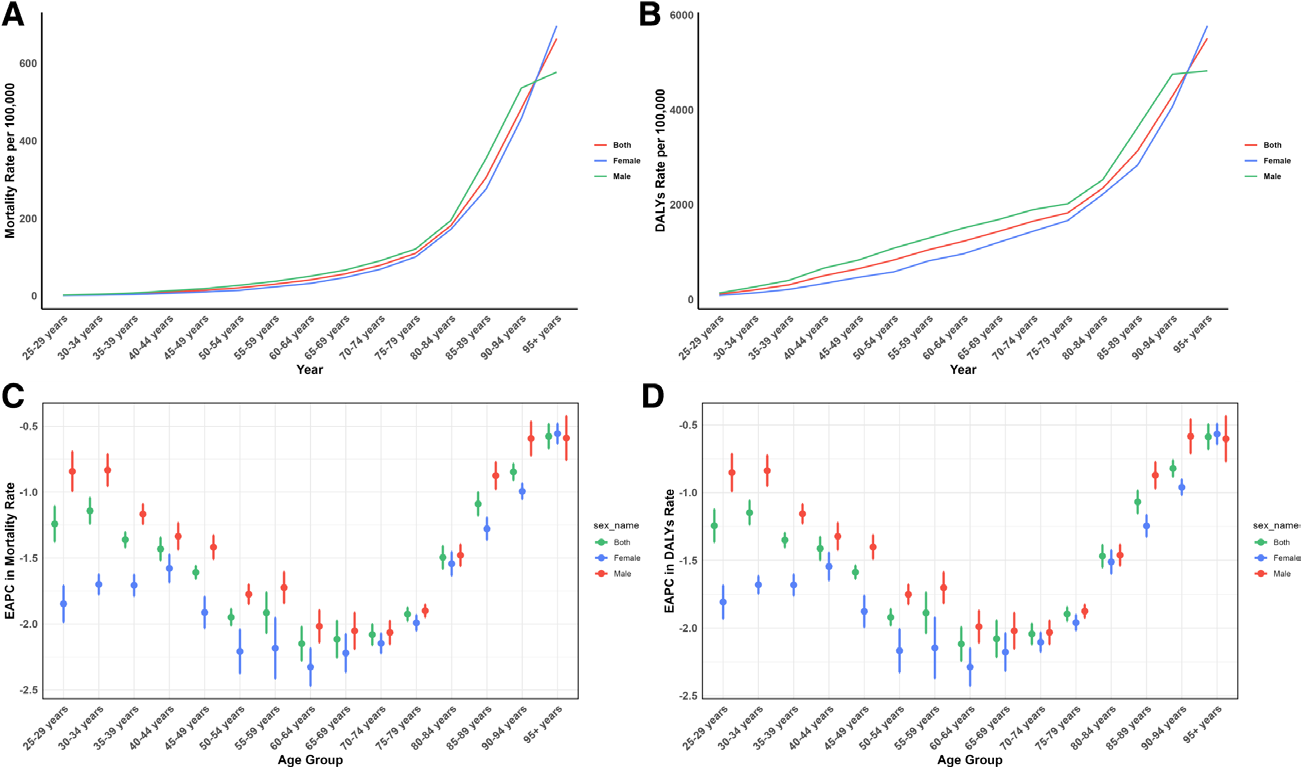
Age-specific rates of global deaths (A) and DALYs (B) of CVD attributable to diet low in fruits, by sex, in 2021 and the corresponding EAPC of global deaths (C) and DALYs (D) from 1990 to 2021. CVD = cardiovascular diseases, DALYs = disability-adjusted life years, EAPC = estimated annual percentage change.

Figure 3 shows the global age-specific mortality and DALY rates for CVDs in 2021, along with trends from 1990 to 2021. The graph displays a J-shaped curve, indicating an increase in mortality and DALY rates for those under 75 years, with a sharp rise in individuals aged 75 to 95 and older. Across all age groups, males had higher mortality rates linked to low fruit consumption, reflecting a greater CVD burden in men. A similar pattern was observed in DALY rates. Additionally, the decline in mortality and DALY rates from 1990 to 2021 was more significant in females under 70 compared to males.

**Figure 4.**
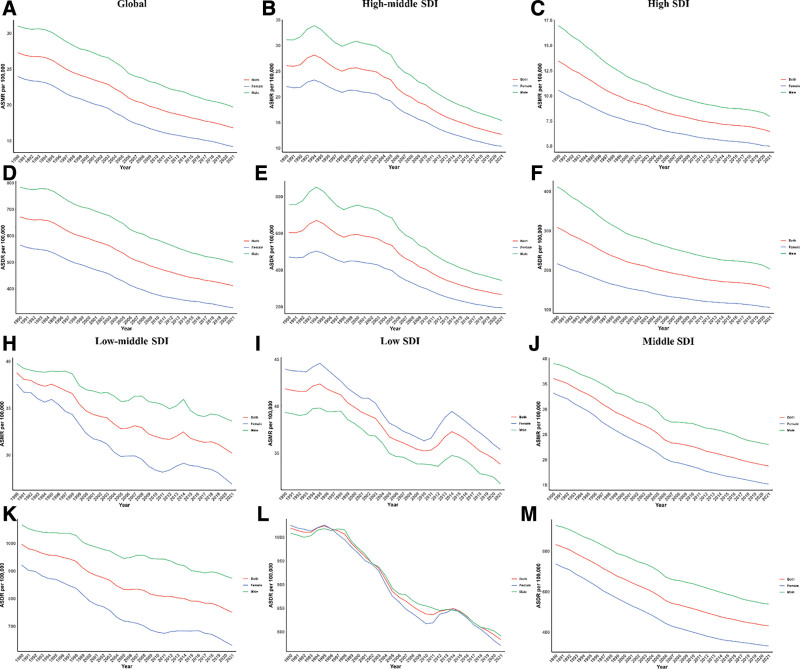
Sex disparity in CVD attributable to diet low in fruits across SDI regions. CVD = cardiovascular diseases, SDI = socio-demographic index.

In all SDI categories, males consistently showed higher mortality and DALY rates, except in low-SDI regions, underscoring a persistent gender disparity across regions. The gap between male and female rates narrowed in High and High-middle SDI regions (Fig. 4).

### 3.3. Association with the socio-demographic index

**Figure 5.**
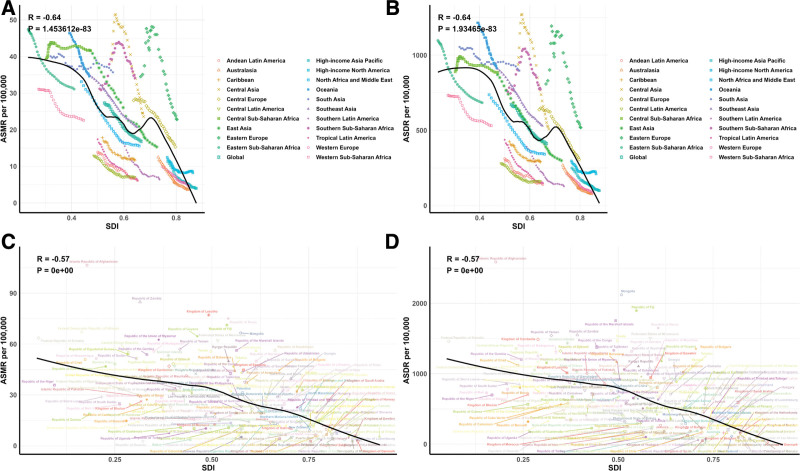
Correlations between ASMR (A and C) and ASDR (B and D) of CVD attributable to diet low in fruits and SDI at the regional level (A and B) and the national level (C and D). ASDR = age-standardized DALY rate, ASMR = age-standardized mortality rate, CVD = cardiovascular diseases, DALY = disability-adjusted life year, SDI = socio-demographic index.

Figure 5 provides a comparison of actual and predicted age-standardized DALY (ASDR) and mortality rates (ASMR) for CVDs linked to low fruit intake, plotted against SDI values at both regional and national levels from 1990 to 2021. A significant negative correlation (R = −0.64, *P* <.0001) was found between ASDR and increasing SDI, suggesting a greater disease burden in regions with lower SDI. Areas like Eastern Europe, Central Asia, and South Asia exhibited higher-than-expected ASDRs during this time. The trends in observed versus predicted ASMR at the regional level were consistent with the ASDR findings. Figure 5 further illustrates the observed and forecasted ASDR and ASMR for 2021 at the national level, showing a similar negative relationship with SDI, both regionally and nationally.

### 3.4. Forecasts for the mortality, DALYs rate, ASMR and ASDR of CVD attributable to diet low in fruits in global (2022–2050)

**Figure 6.**
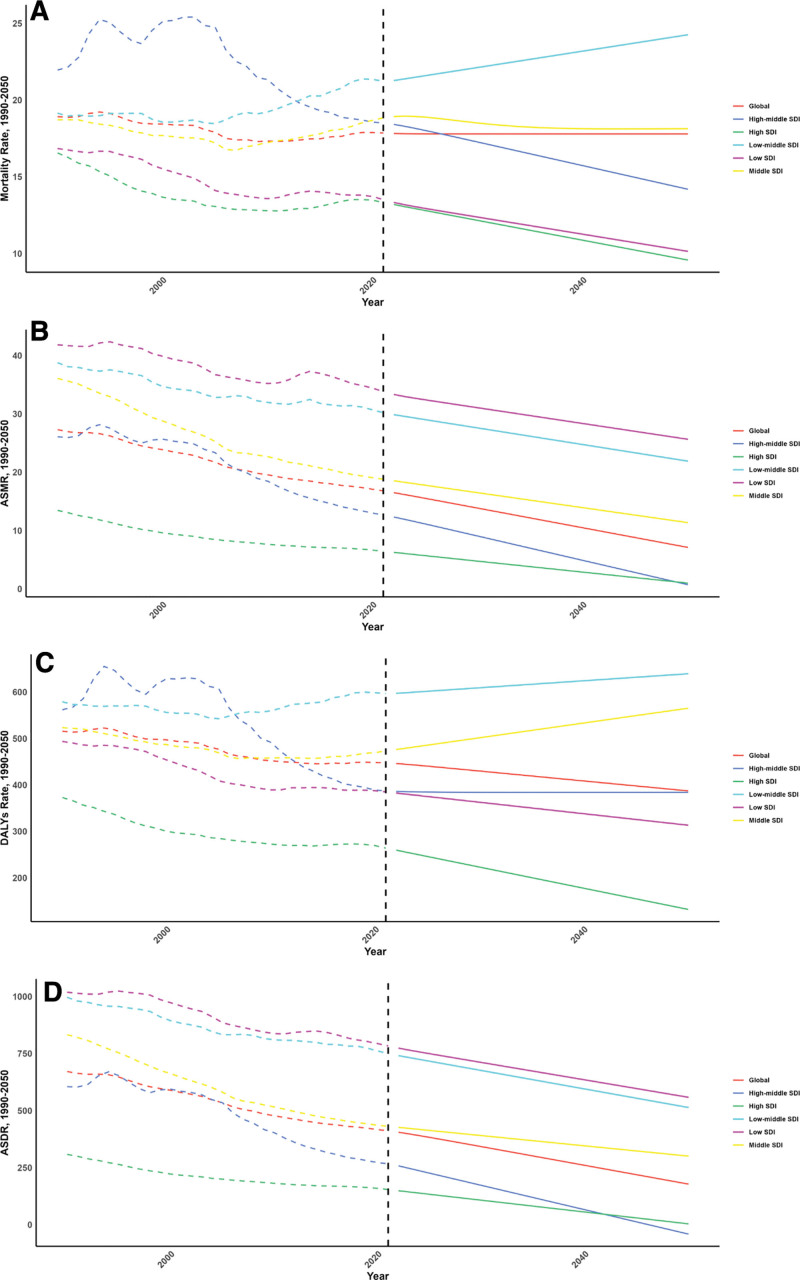
Estimated trends of mortality rate (A), DALYs Rate (B), ASMR (C) and ASDR (D), 1990 to 2050 at the regional level. ASDR = age-standardized DALY rate, ASMR = age-standardized mortality rate, DALYs = disability-adjusted life years.

**Figure 7.**
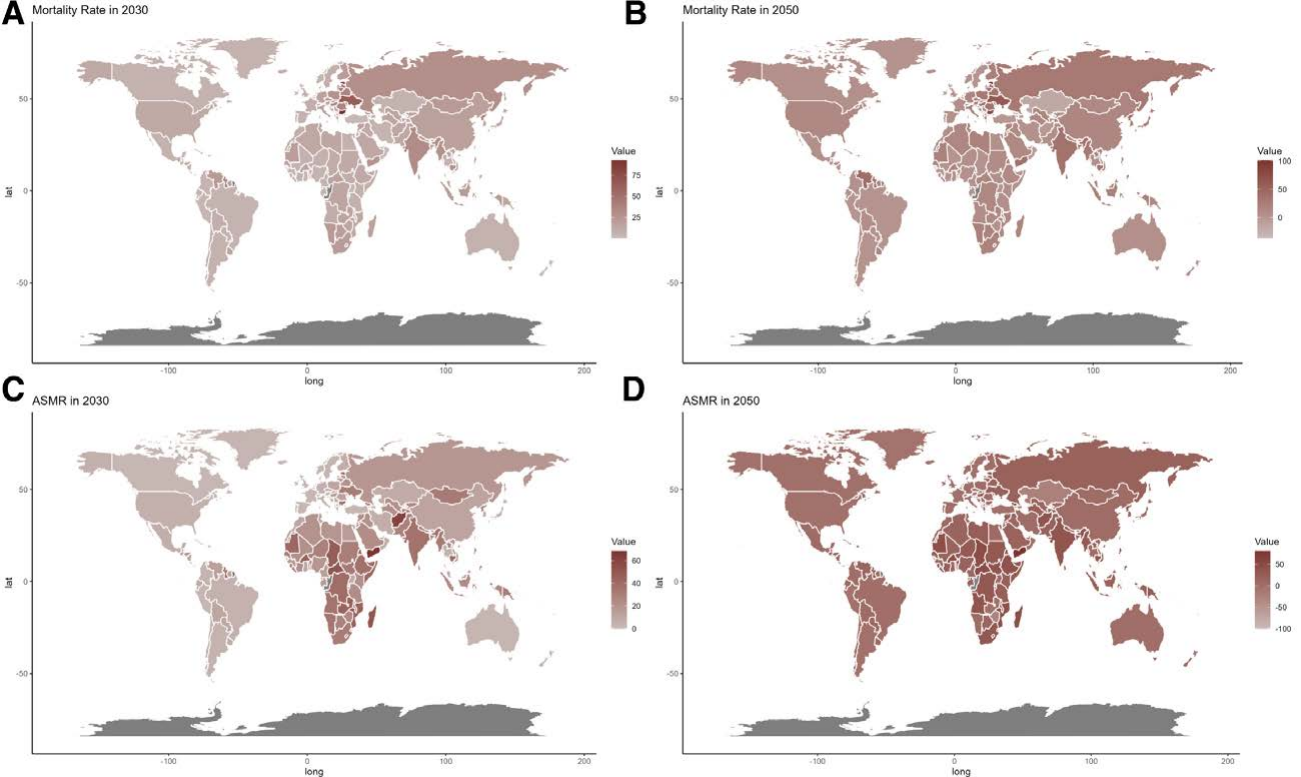
Estimated trends of mortality rate (A and B) and ASMR (C and D) in 2030 (A and C) and 2050 (B and D) at the national level. ASMR = age-standardized mortality rate.

Future projections for ASMR and ASDR related to CVDs from fruit intake are illustrated in Figures 6 and 7. These estimates suggest that most SDI regions will see a decline in CVD burden, except for the low-middle SDI regions, which are expected to face increases in mortality and DALY rates.

At the national level, forecasts indicate that trends will generally remain stable through 2030 and 2050. However, countries in Africa are predicted to experience significantly higher impacts from CVDs related to low fruit intake by both 2030 and 2050.

## 4. Discussion

This study examines the global impact of cardiovascular diseases (CVD) related to low fruit intake from 1990 to 2021, noting a significant decline in such cases over this period. The most affected group was individuals over 75, with men showing higher rates. Projections suggest an increasing CVD burden in lower SDI regions, with African countries expected to experience consistently high levels. The research offers an extensive analysis of how fruit deficiencies influence CVD, along with future trends.

Many previous studies have explored the connection between fruit and vegetable consumption and CVD risk, generally finding a protective effect. For instance, a recent meta-analysis of prospective cohort studies involving 4031,896 participants from 69 countries found that those in the highest consumption category had a 7%, 9%, and 6% lower risk of CVD for total fruit and vegetables, fruit, and vegetables, respectively, compared to those in the lowest category.^[12]^ However, only a few Asian studies have investigated these associations, with mixed results. In agreement with our findings, the China-PAR study reported a 15% reduction in CVD risk among participants in the highest quintile of total fruit and vegetable intake (0.85 [0.77–0.95]).^[21]^ This study also found that higher fruit consumption was associated with an 18% lower CVD risk (0.82 [0.74–0.91]), though no significant association was seen for vegetables. In contrast, Takachi et al found no link between total fruit and vegetable intake and CVD risk among Japanese populations, although they did observe a reduced risk associated with fruit consumption (0.81 [0.67–0.97]).^[19]^ The reasons for these discrepancies remain unclear.

There are several proposed mechanisms through which fruit and vegetable intake may lower CVD risk. In general, fruits and vegetables contain beneficial components such as dietary fiber, plant proteins, vitamins C, minerals, polyphenols, phytoestrogens, and carotenoids, all of which contribute to cardiovascular health.^[42]^ For example, dietary fiber supports gut flora, leading to the production of short-chain fatty acids that help reduce inflammation and platelet aggregation, thereby promoting heart health.^[43]^ Vitamin C, a powerful antioxidant, has been linked to lower risks of CVD conditions like hypertension, coronary heart disease, heart failure, and stroke.^[44]^ Additionally, plant proteins have been associated with a reduced risk of CVD.^[44]^ Polyphenols found in fruits and vegetables, such as phenolic acids and flavonoids from sources like soybeans and grapes, are believed to support heart health through anti-inflammatory, antioxidant, and gut microbiota-regulating pathways.^[44]^

To further enhance the understanding of how fruit consumption affects cardiovascular health, future studies should examine the specific types of fruit and preparation methods. Certain fruits, like berries and citrus fruits, contain bioactive compounds such as flavonoids and vitamin C, which may have a stronger protective effect against CVD. Additionally, the method of preparation – whether fresh, juiced, or processed – could impact the retention of beneficial nutrients. Investigating how different fruits and their combinations with other dietary components affect heart health could provide valuable insights for public health recommendations and individualized dietary strategies to mitigate CVD risks.

To address the global burden of cardiovascular diseases (CVD) attributed to low fruit intake, several targeted interventions are recommended. Public education campaigns should emphasize the cardiovascular benefits of specific fruits and promote their affordable, culturally appropriate consumption. Economic interventions such as fruit subsidies and price reductions can make healthy food choices more accessible, particularly in low-income regions. Schools and workplaces can integrate fruit-focused initiatives into their programs, while governments should update national nutritional guidelines to include clear targets for fruit intake. Lastly, collaborations with the private sector can ensure the availability of affordable and nutritious fruit products, further supporting CVD prevention.

In addition to gender differences, regional cultural and dietary behaviors play a significant role in influencing fruit consumption and the burden of cardiovascular diseases (CVD). For example, in regions with limited access to fruits or where cultural preferences favor other foods, such as grains or root vegetables, fruit consumption may be lower, contributing to higher CVD rates. Regional dietary behaviors, socioeconomic factors, and public health campaigns can further influence gender-specific trends in fruit intake. Understanding these factors helps contextualize the global disparities in CVD burden and highlights the need for targeted interventions that address regional and cultural differences in dietary habits.

To reduce the global burden of cardiovascular diseases linked to low fruit intake, public health authorities should implement a range of strategies. These include public awareness campaigns, economic incentives to make fruits more affordable, and the integration of fruit consumption recommendations into national dietary guidelines. Schools and workplaces can serve as key environments to promote healthy eating habits, and monitoring and evaluation frameworks should be established to assess the success of these interventions. Collaboration between public health organizations, the private sector, and civil society will also be critical to ensuring the widespread adoption of healthier dietary practices.

Future research should focus on key areas to deepen our understanding of the relationship between fruit intake and cardiovascular disease (CVD). Specifically, studies on local fruit accessibility, socioeconomic barriers, and cultural factors influencing dietary habits are essential to address regional disparities in fruit consumption. Additionally, research on the role of the gut microbiota in mediating the cardiovascular benefits of fruits could offer new insights into the mechanisms at play. Long-term longitudinal studies and randomized controlled trials examining fruit consumption interventions will be vital in providing robust evidence for public health strategies aimed at reducing CVD burden.

This study has several limitations. One major issue is the lack of data from certain countries, which may have introduced bias into the analysis. Additionally, the data on total and daily fruit consumption lacked detail. The limited variety of fruits considered also restricted our ability to comprehensively assess CVD risk. Furthermore, there were data gaps concerning mortality and DALY trends from 1990 to 2021. Importantly, data for individuals under 25, a key group with low fruit intake, was unavailable.

## 5. Conclusion

This study provides a thorough evaluation of the global impact of CVD due to low fruit intake from 1990 to 2021, revealing a notable reduction over this period. The most affected group included individuals over 75, particularly males. Projections indicate increasing mortality and DALY rates in low-SDI regions. At the national level, African countries are expected to face persistently high CVD burdens related to low fruit consumption through 2030 and 2050.

**Figure 8.**
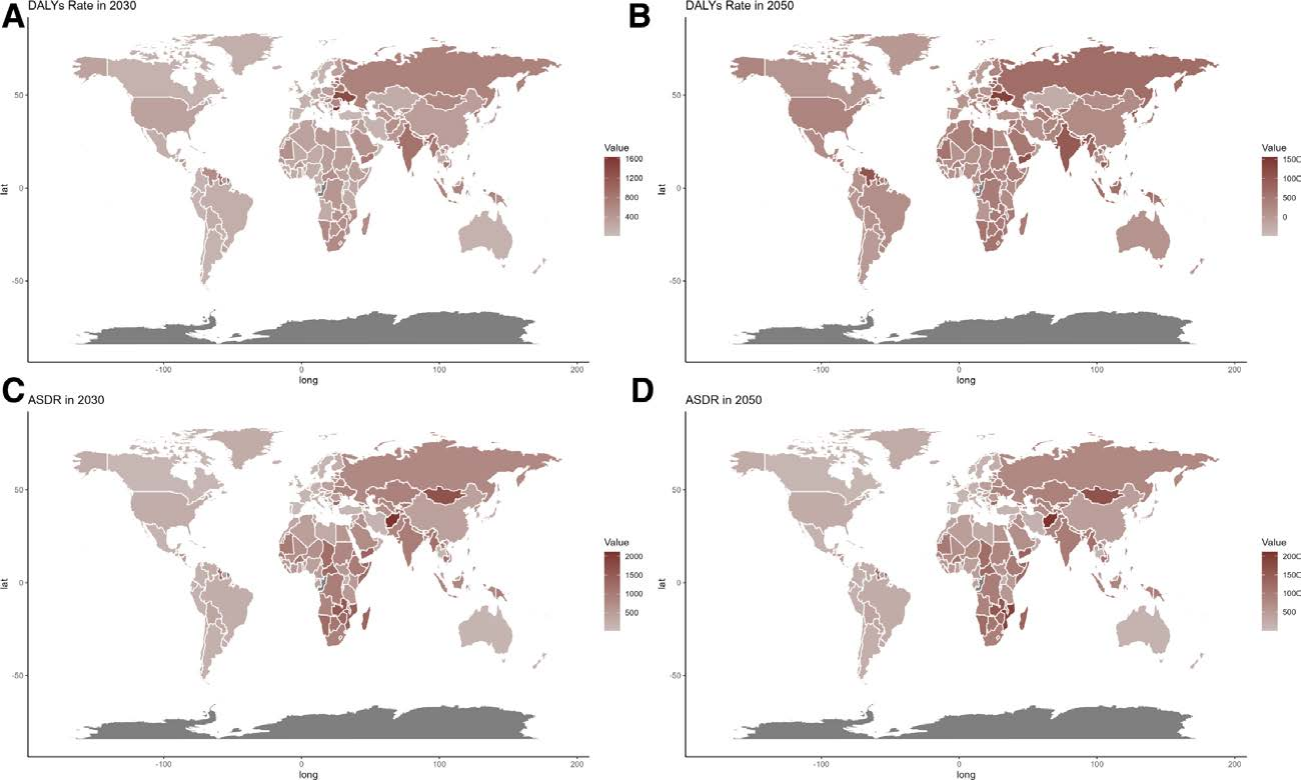
Estimated trends of DALYs rate (A and B) and ASDR (C and D) in 2030 (A and C) and 2050 (B and D) at the national level. ASDR = age-standardized DALY rate, DALYs = disability-adjusted life years.

These findings are crucial for shaping CVD prevention efforts and highlight the importance of proper dietary management (Fig. 8).

## Author contributions

**Conceptualization:** Yu Zuo.

**Data curation:** Yuanxiu Hong, Yu Zuo.

**Formal analysis:** Yuanxiu Hong, Yu Zuo.

**Investigation:** Yuanxiu Hong, Yu Zuo.

**Methodology:** Yu Zuo.

**Project administration:** Yu Zuo.

**Resources:** Yu Zuo.

**Software:** Yu Zuo.

**Supervision:** Yu Zuo.

**Validation:** Yu Zuo.

**Visualization:** Yu Zuo.

**Writing – original draft:** Yuanxiu Hong, Yuzhe Kong, Yu Zuo.

**Writing – review & editing:** Yu Zuo.

## Supplementary Material


