# Supplementary material for: Global burden of cardiovascular diseases attributable to diet low in fruits from 1990 to 2021 and forecasting the future trends: A population-based study
Source: Medicine (Baltimore). 2026 Jan 16;105(3):e44189. doi: 10.1097/MD.0000000000044189 (PMC12826274; doi:10.1097/MD.0000000000044189)
Supplement: Supplementary file 1 [file medi-105-e44189-s001.docx]

Table S1 Global and regional deaths and DALYs of CVD Attributable to Diet Low in Fruits in 1990 and 2021 in 204 nations

| Location | Deaths Number in 1990 | Deaths Number in 2021 | ASMR in 2021 | DALY Number in 1990 | DALY Number in 2021 | ASDR in 2021 |
| --- | --- | --- | --- | --- | --- | --- |
| People's Republic of China | 272214.1022 (403330.0080, 121549.5317) | 277125.8157 (433158.2810, 124233.8291) | 15.3950 (23.7835, 7.2204) | 7201381.3741 (10737994.7512, 2769041.9243) | 5796241.0706 (9255719.3482, 2245055.4803) | 297.8711 (469.3863, 119.1446) |
| Democratic People's Republic of Korea | 3813.8951 (6142.9298, 1559.8930) | 8539.8782 (13195.9274, 3452.5973) | 28.5610 (44.0425, 12.3809) | 103521.9774 (169965.7847, 35299.0276) | 213695.3679 (338880.3886, 75514.2647) | 658.6755 (1035.6389, 239.5895) |
| Taiwan (Province of China) | 1965.2599 (2850.5304, 949.0971) | 2120.4014 (2849.0580, 1419.8052) | 4.8105 (6.4504, 3.1740) | 51098.7067 (75406.3543, 20429.8467) | 42777.1471 (58112.5110, 26870.6729) | 106.9629 (145.7936, 65.0239) |
| Kingdom of Cambodia | 2465.2432 (3711.9595, 1017.8233) | 4633.6130 (7071.9033, 2133.0344) | 42.5129 (63.6950, 21.0873) | 73229.1707 (109782.8015, 27432.6317) | 126805.5082 (193529.4419, 53809.6593) | 983.6182 (1488.5257, 441.7360) |
| Republic of Indonesia | 36915.8739 (58776.0433, 11070.5897) | 59464.6802 (93984.4837, 19889.3524) | 27.2359 (42.7910, 10.1321) | 1220225.5027 (1935256.2016, 340206.6643) | 1808263.2653 (2852041.2291, 556302.9192) | 682.9995 (1074.0541, 224.6183) |
| Lao People's Democratic Republic | 1457.0572 (2213.8392, 531.7822) | 1216.0167 (1868.9621, 505.6246) | 29.4695 (44.6516, 13.0612) | 44994.4734 (69176.8971, 14802.2827) | 35802.4598 (56228.2636, 14370.5590) | 695.3899 (1071.4558, 288.1158) |
| Malaysia | 2390.9893 (3924.8062, 573.2089) | 2938.9916 (4944.0285, 690.2679) | 11.0161 (18.4867, 2.6930) | 70344.4215 (114108.3612, 15569.4642) | 85366.7819 (141968.7808, 19018.6013) | 283.7439 (474.5817, 64.9491) |
| Republic of Maldives | 30.8997 (50.1873, 8.1862) | 36.2663 (56.3266, 13.3252) | 10.9545 (16.9003, 4.3453) | 1024.5920 (1653.7989, 236.8821) | 1103.5891 (1726.1781, 349.1420) | 252.8521 (390.0123, 89.9045) |
| Republic of the Union of Myanmar | 16192.0565 (26477.3560, 5338.1470) | 15702.3844 (24657.1898, 5310.0914) | 34.8136 (54.3004, 13.1667) | 503254.3829 (822094.2087, 152110.9321) | 435191.5385 (686554.1742, 135019.8424) | 852.5987 (1333.6652, 276.9855) |
| Republic of the Philippines | 6935.1560 (9802.2591, 3765.6533) | 17615.3024 (24652.2894, 10064.1277) | 23.2218 (32.3731, 13.7230) | 210335.8204 (299365.6984, 103700.3993) | 515605.1772 (752305.8348, 263486.6738) | 575.5891 (820.5525, 314.8750) |
| Democratic Socialist Republic of Sri Lanka | 3323.8415 (4830.4809, 1582.0339) | 4680.4781 (7786.6544, 1800.7687) | 18.6283 (30.7544, 7.2147) | 92241.6196 (135369.5252, 39193.8210) | 116874.0726 (194945.8971, 41152.7056) | 442.6409 (736.4372, 155.6869) |
| Kingdom of Thailand | 3801.3755 (6325.1638, 913.6812) | 4275.7210 (7164.1380, 1396.9096) | 4.1258 (6.9300, 1.3151) | 116400.7120 (190721.8087, 24119.9894) | 117985.6517 (198608.2282, 29961.3702) | 121.5506 (205.2193, 28.4957) |
| Democratic Republic of Timor-Leste | 125.6360 (196.2980, 46.4524) | 367.0916 (585.8746, 122.8339) | 47.1295 (75.5457, 17.2040) | 4034.7134 (6320.3099, 1312.7292) | 9906.9275 (15739.9370, 3025.1694) | 1126.7096 (1795.4456, 362.7070) |
| Socialist Republic of Viet Nam | 11909.4996 (18212.3683, 5148.1486) | 16918.9624 (27217.4531, 6497.9989) | 18.6522 (29.2876, 7.8555) | 308131.5563 (476241.9857, 122692.0821) | 440789.0834 (719657.8662, 158659.8803) | 430.7753 (696.3293, 162.1343) |
| Republic of Fiji | 254.7655 (399.4264, 81.9179) | 350.5564 (580.7997, 108.6935) | 50.2964 (81.8587, 17.6115) | 8709.6840 (13520.3184, 2629.6936) | 10804.5667 (17785.3055, 3115.4533) | 1299.4923 (2136.8930, 401.5066) |
| Republic of Kiribati | 13.8723 (21.9115, 4.1434) | 23.8227 (39.4195, 7.0846) | 32.8511 (53.7670, 11.4286) | 482.2541 (760.9126, 131.2102) | 846.0818 (1411.4774, 222.7643) | 948.0809 (1564.1467, 275.6168) |
| Republic of the Marshall Islands | 9.8887 (14.9066, 3.6722) | 18.6107 (29.7045, 7.3621) | 54.9701 (85.0185, 24.1939) | 315.9412 (474.1706, 111.1283) | 649.9203 (1046.6253, 237.2725) | 1499.2722 (2373.5741, 606.1948) |
| Federated States of Micronesia | 31.6933 (46.5717, 11.7138) | 38.4975 (62.7717, 15.6216) | 55.5219 (87.0603, 25.7851) | 988.7651 (1483.3195, 326.6590) | 1258.9616 (2083.0652, 464.6433) | 1495.4377 (2437.7388, 592.4694) |
| Independent State of Papua New Guinea | 702.0242 (1125.9461, 241.4128) | 1640.2882 (2620.8825, 569.9842) | 33.3858 (51.6684, 12.5316) | 23063.7298 (36953.9618, 7615.7200) | 54254.9835 (86557.3523, 18440.4571) | 868.9652 (1381.1893, 308.2266) |
| Independent State of Samoa | 35.6678 (54.8531, 14.6812) | 50.5571 (79.3364, 21.3051) | 37.2357 (57.5165, 16.0848) | 1027.9840 (1641.7120, 376.0033) | 1480.4419 (2351.9256, 577.4434) | 959.1076 (1516.3027, 388.0914) |
| Solomon Islands | 78.1448 (128.6423, 20.9245) | 169.4070 (277.8772, 56.2717) | 50.7193 (82.3658, 17.9299) | 2532.7926 (4182.4743, 635.8450) | 5597.2686 (9172.9816, 1766.8604) | 1334.5756 (2176.5814, 439.2501) |
| Kingdom of Tonga | 10.8069 (17.5339, 3.4950) | 13.2491 (22.3476, 4.3500) | 16.7170 (28.2965, 5.5668) | 321.9218 (519.3415, 93.7471) | 366.4562 (623.0011, 112.2578) | 440.7125 (748.7179, 136.5117) |
| Republic of Vanuatu | 19.1744 (31.9137, 4.9475) | 58.1428 (94.9806, 17.3355) | 32.6425 (53.4782, 10.2496) | 686.7029 (1141.9613, 163.3725) | 2050.2339 (3288.0167, 574.1233) | 946.8848 (1527.6902, 278.7626) |
| Republic of Armenia | 571.5879 (883.2019, 233.9149) | 362.9278 (562.1449, 188.0947) | 8.5553 (13.1001, 4.5306) | 13673.8099 (21283.2320, 4924.8509) | 6762.8442 (10073.9861, 3250.6732) | 163.4747 (242.0025, 76.1423) |
| Republic of Azerbaijan | 1424.1783 (2186.0409, 549.4026) | 1339.3008 (2099.7233, 578.9214) | 15.9418 (24.9797, 7.3070) | 37576.7635 (57546.3932, 13307.5986) | 32973.7620 (52117.3556, 13591.2806) | 330.1262 (513.5475, 139.5030) |
| Georgia | 1619.1910 (2748.8541, 385.7536) | 1745.9636 (2464.7115, 999.5938) | 27.7025 (39.3860, 15.6452) | 40228.6099 (66857.1905, 8961.5532) | 34058.7519 (49582.8966, 17032.7753) | 596.5497 (867.3324, 281.9138) |
| Republic of Kazakhstan | 5250.0024 (8515.5597, 1382.2307) | 3106.5436 (5166.2179, 849.0172) | 21.1953 (35.4952, 5.8165) | 145474.5074 (231871.4921, 39323.6901) | 76044.2040 (125258.7269, 20184.3379) | 437.1872 (721.5781, 119.0925) |
| Kyrgyz Republic | 1178.2636 (1952.7102, 344.6287) | 1391.7769 (2126.3989, 525.8702) | 33.6421 (51.8263, 12.8307) | 32069.5916 (51778.7909, 8897.8170) | 35315.1840 (53783.8027, 12953.0440) | 719.0676 (1102.4869, 269.0493) |
| Mongolia | 717.2765 (1190.5758, 126.7591) | 886.4652 (1464.5753, 172.5063) | 44.4255 (74.0723, 9.4917) | 19256.0399 (31419.7025, 3219.5512) | 26068.2834 (41974.6145, 4860.5834) | 1027.8523 (1683.7101, 205.7983) |
| Republic of Tajikistan | 1356.4410 (2010.3210, 614.2606) | 1763.9327 (2655.8692, 754.5644) | 38.5103 (58.2883, 17.3602) | 33438.3816 (50337.4797, 14116.3602) | 46425.3482 (70981.1061, 19118.0388) | 791.0292 (1191.3847, 332.6294) |
| Turkmenistan | 648.5388 (1033.1408, 209.5802) | 799.8325 (1336.8798, 288.4730) | 21.5337 (35.7458, 8.0682) | 17973.5822 (28580.4092, 5492.7250) | 22394.6906 (37364.4981, 7639.6534) | 520.6480 (861.5475, 182.2263) |
| Republic of Uzbekistan | 4261.9552 (6769.8491, 1344.9189) | 5312.1028 (8190.9019, 1985.1548) | 24.3819 (37.9130, 9.7561) | 111763.9935 (175784.5666, 34358.5724) | 139090.9576 (215926.8902, 51493.6137) | 521.0146 (798.5527, 192.8409) |
| Republic of Albania | 367.2077 (613.1051, 98.0485) | 229.3396 (417.4545, 75.2033) | 5.9446 (10.6722, 1.9708) | 8743.0904 (14452.2066, 2032.7126) | 3827.0392 (6741.9907, 1211.6131) | 98.2730 (172.7821, 31.1563) |
| Bosnia and Herzegovina | 1010.8946 (1591.1643, 323.4607) | 613.8081 (962.9274, 295.0410) | 9.8744 (15.4536, 4.7546) | 26984.4027 (42413.2735, 8142.7349) | 11522.7400 (18248.7439, 5371.0104) | 195.3726 (308.3058, 87.8407) |
| Republic of Bulgaria | 3116.6054 (4759.5632, 1261.3822) | 6949.4823 (9067.1980, 4800.7899) | 50.1473 (65.5821, 34.6039) | 74638.6259 (113320.4760, 28495.1624) | 129208.8091 (171418.5811, 86703.3408) | 992.3128 (1334.0400, 634.5158) |
| Republic of Croatia | 1323.4591 (2034.9539, 558.9817) | 758.8394 (1197.7161, 371.5140) | 8.0026 (12.4373, 3.8398) | 28545.7375 (44585.2227, 10109.9862) | 12368.7842 (18974.4776, 5530.7434) | 145.2217 (224.2487, 62.2690) |
| Czech Republic | 2924.1166 (5026.4603, 696.8983) | 2472.5928 (3861.1841, 1103.4556) | 10.8912 (17.0859, 4.7723) | 68324.1214 (113632.8331, 15697.6509) | 44020.3024 (67835.5557, 18430.6884) | 212.8121 (327.4742, 86.2273) |
| Hungary | 3213.1135 (4969.5698, 1224.6042) | 3341.8391 (4761.2938, 1800.6972) | 16.2503 (23.2909, 8.5455) | 76967.9981 (119568.7913, 26474.8095) | 62488.2183 (89219.5978, 30979.5456) | 339.4364 (493.0903, 163.9955) |
| North Macedonia | 371.9889 (556.3880, 186.5237) | 407.1057 (626.0919, 207.7717) | 17.3665 (26.3656, 9.6340) | 8882.6944 (13529.4078, 3479.8750) | 8246.9078 (12844.2960, 3845.3097) | 294.1517 (447.0230, 149.4363) |
| Montenegro | 60.5631 (105.8354, 14.1364) | 59.2820 (109.5758, 16.4470) | 7.2837 (13.4144, 2.2644) | 1500.7865 (2543.9367, 299.1842) | 1113.6563 (2014.2957, 282.8764) | 129.0538 (233.9827, 34.5042) |
| Republic of Poland | 13630.3305 (21771.3213, 4405.8302) | 10114.2703 (14943.7127, 5421.9127) | 13.3340 (19.5610, 7.0157) | 340387.5508 (540845.1906, 101510.1042) | 185008.7676 (275062.1019, 92138.0921) | 267.9383 (398.4193, 128.4765) |
| Romania | 7552.0678 (11306.7243, 3111.1893) | 6470.9220 (9114.3299, 3621.7906) | 16.4140 (23.1556, 9.1021) | 173078.4584 (264241.8803, 63649.8060) | 117732.5924 (170213.8755, 59439.7683) | 334.2162 (485.7457, 160.1512) |
| Republic of Serbia | 2049.9575 (3123.6935, 884.6535) | 1632.5821 (2439.7090, 873.7398) | 9.7265 (14.4777, 5.0952) | 46851.2964 (73748.0549, 16740.5788) | 27956.6724 (42292.3340, 13578.0575) | 177.4608 (271.4868, 81.5167) |
| Slovak Republic | 1858.8112 (3039.3749, 506.5537) | 1494.0195 (2377.0058, 560.5331) | 15.9173 (25.3072, 5.9490) | 44313.9024 (70488.8092, 11666.2429) | 29421.7921 (47100.4292, 10580.5058) | 322.7844 (514.3509, 114.4845) |
| Republic of Slovenia | 225.6036 (332.9295, 115.2829) | 278.0725 (393.2758, 175.7571) | 4.9421 (6.9938, 3.1881) | 5040.9400 (7442.9413, 2159.0066) | 3853.6995 (5288.9657, 2467.3663) | 79.9357 (109.4820, 48.5475) |
| Republic of Belarus | 4020.8770 (6725.7884, 967.0643) | 3536.7717 (6180.4510, 754.1869) | 22.1831 (38.2911, 4.7500) | 95410.7115 (154595.7975, 23139.4468) | 75580.0222 (129210.4869, 16576.1130) | 499.3310 (850.8486, 109.0754) |
| Republic of Estonia | 731.6075 (1190.8102, 212.9464) | 981.0042 (1284.5795, 700.2214) | 29.6487 (38.3970, 21.3108) | 16674.7057 (26543.3528, 5097.0001) | 14107.3208 (18321.7898, 10046.1564) | 485.7635 (623.4265, 348.5038) |
| Republic of Latvia | 1205.9295 (2050.8884, 263.6208) | 919.5786 (1399.1291, 422.6485) | 21.2611 (32.5737, 9.3177) | 28001.2798 (46537.7541, 6135.1899) | 16983.0503 (26167.5192, 7272.2557) | 456.7582 (701.1087, 186.8404) |
| Republic of Lithuania | 1275.0801 (2141.0319, 296.0888) | 984.2080 (1630.1687, 351.5302) | 15.6055 (25.5164, 5.4903) | 28634.7547 (46836.0137, 6614.7851) | 17801.2846 (29321.5961, 6116.8762) | 330.9533 (536.4299, 109.6487) |
| Republic of Moldova | 1052.2744 (1835.6936, 198.1699) | 1596.2137 (2372.3998, 734.6163) | 26.7237 (39.7775, 12.3156) | 25807.2820 (43764.2236, 4889.1042) | 33348.5687 (49540.5370, 14451.2132) | 575.0016 (853.7187, 245.9709) |
| Russian Federation | 57831.7295 (96423.3909, 14322.3861) | 43529.8547 (72435.0035, 13116.0989) | 18.5751 (30.9309, 5.5457) | 1479448.9234 (2399596.1190, 366858.6603) | 990125.3161 (1635803.9726, 284382.3154) | 442.9589 (730.9194, 123.8849) |
| Ukraine | 22277.9969 (37233.8642, 5527.2592) | 27789.9400 (48788.7562, 7323.7187) | 35.8344 (62.7656, 9.4554) | 500920.3371 (824872.0967, 124203.4222) | 564537.7180 (976737.2018, 147640.5899) | 766.1759 (1313.2069, 199.1472) |
| Brunei Darussalam | 24.7605 (38.2683, 9.2655) | 47.6247 (72.7809, 18.9375) | 16.7648 (24.8888, 8.3859) | 787.6767 (1220.1911, 246.8498) | 1517.9073 (2340.4882, 536.4353) | 381.8947 (578.9854, 151.8433) |
| Japan | 15380.7253 (24091.3335, 5922.2809) | 17786.3336 (26484.5309, 8565.1809) | 4.0895 (6.3149, 1.5485) | 346631.8480 (551206.6133, 105352.3832) | 313290.3493 (488463.7339, 119202.6819) | 111.7704 (177.2541, 32.4365) |
| Republic of Korea | 4580.7009 (7282.9271, 1439.4844) | 4493.1389 (6677.7480, 2503.2942) | 5.1867 (7.6391, 2.8859) | 132224.1533 (212680.1426, 34362.8190) | 77594.4178 (115797.7472, 36740.6056) | 90.0352 (134.6898, 42.8988) |
| Republic of Singapore | 306.6890 (462.9498, 147.1131) | 305.2203 (402.1818, 204.6112) | 3.6458 (4.8033, 2.4546) | 8550.7630 (13099.6809, 3467.7176) | 7096.6461 (9303.4928, 4344.5384) | 83.4877 (109.5166, 51.3270) |
| Australia | 2481.8039 (4243.8572, 651.5023) | 1892.4149 (2957.6469, 767.2979) | 3.7625 (5.8859, 1.4651) | 53547.0612 (89898.9477, 13086.7091) | 34147.5944 (53714.4002, 12384.2204) | 80.0401 (123.8247, 27.2682) |
| New Zealand | 337.7861 (560.4487, 103.6501) | 280.3430 (459.5694, 116.5732) | 3.1879 (5.2492, 1.2743) | 7782.3655 (12579.6604, 2115.9475) | 5444.9829 (8744.4764, 1953.9625) | 69.7488 (111.6849, 23.6213) |
| Principality of Andorra | 2.6476 (4.2219, 1.2085) | 5.6431 (9.1361, 2.9645) | 3.1026 (5.0701, 1.6195) | 61.1098 (99.9715, 24.2325) | 90.7250 (143.8818, 46.6428) | 56.4253 (90.1940, 27.4634) |
| Republic of Austria | 1015.1965 (1591.3074, 461.2616) | 986.6008 (1446.9383, 590.6489) | 4.1742 (6.0190, 2.5149) | 19844.7219 (30976.6537, 7796.6279) | 13542.4650 (19178.7813, 8024.7350) | 67.6702 (97.0356, 37.3224) |
| Kingdom of Belgium | 1629.7782 (2730.2216, 450.1228) | 750.5521 (1211.6490, 291.0056) | 2.6986 (4.4048, 0.9754) | 34313.0753 (57239.8218, 8384.5334) | 12744.4296 (20676.8569, 4485.9071) | 57.1842 (92.6705, 18.0025) |
| Republic of Cyprus | 66.6457 (101.5040, 32.1667) | 107.3419 (157.5688, 56.6060) | 6.9533 (10.1404, 3.8331) | 1276.1295 (1899.2791, 578.6282) | 1896.3172 (2802.5820, 866.9755) | 108.3349 (157.7555, 54.2284) |
| Kingdom of Denmark | 1289.3198 (2230.8667, 297.5748) | 271.6545 (446.4861, 114.5030) | 2.0790 (3.4158, 0.8230) | 26506.6711 (44950.4642, 5759.2586) | 4637.5725 (7689.1839, 1635.5659) | 42.0788 (69.6253, 13.2563) |
| Republic of Finland | 1265.9721 (2115.1018, 328.6440) | 1329.8589 (1932.3578, 702.5402) | 8.6927 (12.6343, 4.5174) | 28553.8930 (46817.0889, 6934.3059) | 20483.7812 (30038.6164, 10339.4576) | 164.6828 (243.0902, 77.7994) |
| French Republic | 7070.3047 (10977.1925, 2994.3440) | 6818.6931 (10103.3168, 3678.5592) | 3.6520 (5.4178, 1.7918) | 133936.8137 (209013.1073, 48278.1679) | 109495.1659 (161281.8488, 53129.2561) | 77.3684 (115.2296, 31.1480) |
| Federal Republic of Germany | 13893.5976 (21372.6694, 5936.0835) | 15137.8550 (21155.2828, 9130.9917) | 6.4128 (8.9402, 3.7230) | 269335.0514 (417335.5594, 106483.8410) | 229109.3004 (323128.6310, 129005.3815) | 116.7624 (167.8702, 58.7967) |
| Hellenic Republic | 430.1716 (697.7661, 169.3644) | 1007.2051 (1545.2206, 581.7822) | 3.1078 (4.7200, 1.7381) | 8300.8040 (13399.1906, 2682.9788) | 13904.8695 (21034.7530, 7587.3071) | 56.4264 (85.5122, 28.1913) |
| Republic of Iceland | 42.6552 (70.3842, 12.1143) | 28.9884 (46.3143, 12.9123) | 4.3323 (6.8898, 1.8444) | 886.4991 (1438.6187, 226.8938) | 490.5256 (772.7655, 188.5606) | 86.6250 (137.2835, 30.5790) |
| Ireland | 830.7875 (1393.1296, 200.0523) | 264.2478 (419.4642, 94.9310) | 3.1880 (5.0800, 1.1231) | 18259.9387 (30150.6335, 4161.8822) | 4891.7252 (7984.7136, 1558.5566) | 64.4577 (106.0481, 19.6779) |
| State of Israel | 105.7667 (181.5991, 37.9107) | 125.5583 (200.6736, 56.9218) | 0.9082 (1.4496, 0.3899) | 2229.8545 (3747.8905, 648.5360) | 2301.3676 (3661.7534, 855.6076) | 18.9872 (30.4591, 6.3612) |
| Republic of Italy | 4453.1967 (6376.3302, 2416.9338) | 10954.3559 (15033.2119, 6818.9008) | 5.1899 (7.0231, 3.3341) | 84569.3837 (125737.0936, 39172.4656) | 133904.2956 (179222.6556, 88345.6232) | 76.3157 (101.6032, 48.5368) |
| Grand Duchy of Luxembourg | 53.1018 (85.5268, 17.1126) | 42.6988 (64.1205, 21.9887) | 3.5538 (5.3316, 1.7728) | 1132.1754 (1853.2294, 310.8133) | 734.1221 (1127.1749, 328.0477) | 67.9449 (105.1768, 28.1571) |
| Republic of Malta | 50.4063 (81.5834, 15.0627) | 48.2061 (74.5104, 21.6941) | 4.5755 (7.0901, 1.9452) | 1093.7926 (1779.1595, 282.8797) | 851.4030 (1313.7306, 343.2868) | 95.9429 (150.1549, 35.2729) |
| Kingdom of the Netherlands | 1682.2383 (2782.2219, 454.4509) | 1021.6534 (1579.3530, 504.3611) | 2.6518 (4.1393, 1.2651) | 37595.1874 (62062.3206, 9137.8040) | 17214.2253 (27056.6639, 7410.7569) | 51.7083 (81.4921, 20.2819) |
| Kingdom of Norway | 759.4620 (1286.5533, 210.5752) | 266.5024 (427.0895, 119.5087) | 2.2600 (3.6117, 0.9640) | 15447.7962 (25769.0191, 3902.2903) | 4430.0090 (7038.7684, 1674.1966) | 44.4390 (71.3479, 15.2018) |
| Portuguese Republic | 1346.8232 (2213.4568, 481.5728) | 1218.6424 (1804.5226, 690.4949) | 3.9943 (5.9588, 2.1988) | 28710.5164 (47459.8853, 7827.4822) | 18280.1807 (27071.6406, 9078.0846) | 74.9750 (113.6015, 31.9772) |
| Kingdom of Spain | 1735.4408 (2806.4473, 708.7175) | 3222.2583 (4876.6097, 1923.3088) | 2.3501 (3.4934, 1.4321) | 35932.5832 (57468.1051, 11392.3072) | 44309.9906 (65642.0227, 26244.1956) | 40.9853 (60.9436, 21.7334) |
| Kingdom of Sweden | 1851.5016 (3156.3634, 467.7590) | 1187.0612 (1739.8555, 674.6608) | 4.2971 (6.3707, 2.3295) | 35154.7323 (59594.5814, 8031.5512) | 17276.7528 (25780.4502, 9024.8570) | 75.1920 (113.2852, 35.9960) |
| Swiss Confederation | 1021.8668 (1570.6160, 519.1385) | 1277.4823 (1773.3332, 788.1304) | 5.0380 (7.0574, 3.1593) | 18079.1196 (28060.9616, 7516.2612) | 16472.5363 (22835.7762, 10117.9430) | 78.0574 (108.3579, 46.7394) |
| United Kingdom of Great Britain and Northern Ireland | 16372.1434 (27314.7886, 4471.2537) | 7364.3144 (11527.7428, 3037.0825) | 5.3187 (8.3814, 2.1277) | 352150.6097 (579160.0596, 90575.5950) | 141992.4087 (222615.5220, 54303.4768) | 121.1254 (189.4110, 43.9369) |
| Argentine Republic | 5406.4731 (7657.5948, 2799.2606) | 3734.1482 (5188.4820, 2468.7169) | 6.4542 (8.9736, 4.2439) | 129920.3481 (191559.2904, 56757.4719) | 67748.8703 (96617.3348, 41622.1275) | 122.0207 (173.3248, 72.5298) |
| Republic of Chile | 1416.8853 (2211.9727, 552.9587) | 1533.8564 (2169.4969, 897.3193) | 5.9241 (8.3785, 3.4397) | 34575.9867 (53656.3082, 11501.1665) | 32782.7807 (47270.8166, 15542.8663) | 131.7229 (190.2216, 60.3249) |
| Eastern Republic of Uruguay | 612.3800 (958.9748, 221.9641) | 499.3638 (684.9284, 315.0941) | 7.9053 (10.8597, 4.7662) | 14254.5561 (22810.7202, 4234.4903) | 8915.1997 (12635.3307, 4773.7765) | 166.5078 (241.8087, 82.0792) |
| Canada | 2470.4773 (4168.1438, 627.6307) | 2566.4134 (4128.3323, 1121.2237) | 3.3798 (5.3396, 1.4901) | 55394.0136 (91288.3965, 13665.3288) | 52239.2442 (80293.6036, 23775.1496) | 82.0058 (123.3712, 37.0436) |
| United States of America | 41111.8130 (66100.0654, 14872.2425) | 51260.5022 (71273.9886, 29291.7803) | 8.8266 (12.1377, 5.0544) | 930807.3885 (1444667.0503, 331018.4763) | 1145046.3340 (1564064.4481, 633677.2916) | 226.3979 (306.0803, 126.2623) |
| Antigua and Barbuda | 9.9921 (13.5300, 6.2801) | 15.9716 (20.5341, 11.5112) | 17.2212 (22.1440, 12.6343) | 210.2013 (285.3951, 126.1083) | 345.9652 (442.6374, 241.0316) | 336.0206 (427.6167, 239.1906) |
| Commonwealth of the Bahamas | 46.4343 (59.9879, 31.4788) | 100.1141 (132.2182, 71.0270) | 26.9430 (35.1849, 19.3908) | 1298.4045 (1674.2525, 851.3797) | 2555.3066 (3409.3878, 1792.4354) | 614.9553 (812.5533, 436.2541) |
| Barbados | 73.0816 (106.7232, 37.8307) | 70.8200 (104.5230, 37.8967) | 13.9349 (20.6275, 7.4394) | 1510.9399 (2246.3062, 680.7797) | 1458.0251 (2212.4958, 726.3161) | 298.7439 (454.2466, 148.2004) |
| Belize | 4.2589 (5.8912, 2.7849) | 12.9927 (18.1211, 8.7948) | 4.5640 (6.5664, 3.0437) | 106.3028 (147.2887, 61.6226) | 351.9741 (475.7062, 231.6586) | 105.8144 (143.4570, 71.1484) |
| Republic of Cuba | 1177.8381 (1906.4044, 380.8520) | 1711.8081 (2362.2464, 1069.2855) | 8.3151 (11.4471, 5.0956) | 29745.0828 (47122.8255, 8784.3216) | 33497.1349 (46166.5994, 19578.5287) | 176.8538 (244.0521, 99.5877) |
| Commonwealth of Dominica | 3.7686 (5.4892, 2.2847) | 3.3638 (5.4176, 1.8182) | 4.6258 (7.5347, 2.4552) | 70.1235 (96.6852, 46.9775) | 66.2254 (97.3338, 40.1154) | 86.7404 (128.6683, 51.5978) |
| Dominican Republic | 305.7736 (429.5901, 180.6359) | 417.5347 (657.8304, 241.2581) | 4.2054 (6.6335, 2.4006) | 8739.5408 (12510.5173, 4575.9046) | 10857.0454 (16494.1211, 5962.1896) | 103.3650 (156.3531, 58.0037) |
| Grenada | 11.1847 (15.6929, 6.9585) | 13.3144 (18.1992, 8.1966) | 13.2805 (18.2299, 8.3761) | 256.6825 (357.3245, 136.7607) | 324.2871 (448.6635, 188.2118) | 290.6559 (396.9557, 172.8434) |
| Republic of Guyana | 241.9887 (334.2993, 131.3400) | 249.9670 (352.5602, 151.2139) | 42.7648 (58.5680, 26.8013) | 6599.7965 (9390.3842, 3324.7932) | 6788.9218 (9819.3502, 3981.5025) | 1016.5009 (1440.0130, 600.8303) |
| Republic of Haiti | 960.9290 (1499.1826, 378.2030) | 2032.8821 (3305.0897, 778.1071) | 31.2555 (49.5746, 13.8547) | 28876.7907 (45458.9929, 9520.1246) | 61138.5873 (100105.3113, 21489.3878) | 747.7351 (1211.7173, 284.7109) |
| Jamaica | 394.0889 (495.6861, 288.1913) | 523.3616 (723.6285, 337.6871) | 15.7090 (21.4941, 9.8307) | 7881.9654 (10069.1919, 5508.9062) | 11026.9154 (15280.7251, 6781.4174) | 349.6990 (487.7770, 215.3846) |
| Saint Lucia | 11.2947 (15.3482, 7.2078) | 30.2982 (41.3571, 20.5295) | 13.1699 (17.9364, 8.8766) | 262.4088 (362.3609, 150.0764) | 666.0049 (920.7885, 428.0024) | 282.4544 (389.4502, 183.0310) |
| Saint Vincent and the Grenadines | 24.3211 (32.8453, 14.6840) | 29.2777 (37.5584, 20.7979) | 23.9980 (30.4325, 17.2234) | 554.0720 (752.3569, 309.8694) | 603.8418 (771.1903, 427.0375) | 457.4283 (579.9754, 329.2471) |
| Republic of Suriname | 69.7224 (98.7084, 35.1889) | 110.4726 (162.6815, 63.7616) | 18.0994 (26.5115, 10.6364) | 1833.1479 (2654.7606, 834.9461) | 2804.3486 (4212.4804, 1400.8236) | 438.4005 (653.7697, 224.6520) |
| Republic of Trinidad and Tobago | 322.5437 (460.2453, 162.8780) | 328.7018 (503.5227, 162.8489) | 17.7204 (26.8205, 8.8777) | 8143.4093 (11748.5584, 3633.4461) | 8129.3387 (12560.8746, 3707.7166) | 438.9257 (677.4772, 201.3666) |
| Plurinational State of Bolivia | 602.5883 (922.3212, 257.4564) | 1025.8399 (1607.6897, 518.6048) | 12.8753 (19.8297, 6.9803) | 16360.9694 (25561.3181, 5479.0986) | 25463.0258 (40456.6318, 11188.8404) | 276.9962 (435.3147, 127.4895) |
| Republic of Ecuador | 413.1661 (551.1733, 279.6652) | 809.4858 (1175.8049, 506.1359) | 5.5058 (7.9781, 3.5775) | 9373.9059 (12395.5206, 5629.8641) | 17235.2929 (24927.3602, 9915.6581) | 107.2677 (155.8273, 62.6179) |
| Republic of Peru | 1629.7749 (2423.5571, 760.1370) | 1699.9625 (2660.7714, 765.3210) | 4.9473 (7.7037, 2.2545) | 42672.2822 (65078.8543, 15250.4085) | 43027.8287 (67591.4474, 16766.3081) | 121.9475 (191.4679, 48.6751) |
| Republic of Colombia | 2871.1551 (3936.7949, 1581.5173) | 2579.7433 (3920.2279, 1171.2279) | 4.5290 (6.9209, 2.0052) | 73537.8204 (103997.8435, 36758.9142) | 56224.9229 (86596.7914, 20363.9850) | 101.3201 (156.8885, 36.3424) |
| Republic of Costa Rica | 109.9257 (160.0814, 54.2238) | 203.7150 (288.5496, 113.2435) | 3.5988 (5.1163, 2.0128) | 2692.5200 (3998.7166, 1101.3188) | 4586.0459 (6682.2730, 2031.6775) | 83.1778 (121.2324, 35.8747) |
| Republic of El Salvador | 417.2200 (657.0484, 138.7996) | 538.4450 (878.6084, 187.2707) | 8.1457 (13.3764, 2.7465) | 11443.7490 (18031.7147, 3115.8953) | 12523.0489 (21044.7421, 3991.5352) | 199.7066 (338.0494, 63.0234) |
| Republic of Guatemala | 515.2914 (804.7350, 166.2767) | 855.7388 (1381.6109, 289.9696) | 8.8443 (14.0609, 3.3343) | 15573.8369 (24741.6926, 4238.2118) | 21772.9606 (35900.0559, 5789.7036) | 191.1630 (312.4410, 55.2670) |
| Republic of Honduras | 344.6505 (504.8899, 166.1621) | 1211.8528 (1791.4730, 593.8888) | 22.4083 (33.1022, 11.9920) | 9899.1641 (14620.0409, 3990.5353) | 30109.5865 (46109.2787, 13776.9286) | 470.1953 (705.6627, 227.4845) |
| United Mexican States | 3622.9419 (5127.9673, 1918.2509) | 7086.8228 (10850.1759, 3168.4894) | 6.0633 (9.2030, 2.8181) | 89434.9565 (131977.6823, 36874.9253) | 172115.7391 (269134.3274, 63873.8642) | 134.7851 (209.2301, 52.0745) |
| Republic of Nicaragua | 248.5763 (367.4578, 101.7426) | 662.8936 (1016.2179, 272.7905) | 14.9758 (22.8161, 6.3942) | 6635.5667 (9902.2591, 2285.8524) | 16737.4465 (26271.0687, 5930.9637) | 330.7644 (509.3775, 125.3941) |
| Republic of Panama | 150.2067 (244.6706, 48.3630) | 300.1850 (459.1830, 137.2637) | 6.6057 (10.1642, 2.9670) | 3784.4963 (6126.9929, 1057.2715) | 6937.0964 (10774.4907, 2626.5020) | 155.9347 (242.2311, 58.5669) |
| Bolivarian Republic of Venezuela | 1248.4389 (1723.8136, 702.1553) | 3618.7737 (5514.1615, 1719.8706) | 12.5880 (19.2261, 6.0921) | 34729.7778 (49721.8338, 16358.6920) | 88547.4038 (139207.4403, 37274.8070) | 294.6697 (460.5680, 125.5528) |
| Federative Republic of Brazil | 14740.6842 (21471.6066, 6958.3266) | 15018.0658 (20878.7845, 8289.7968) | 6.1331 (8.4781, 3.4627) | 438135.0906 (650783.1792, 174817.6752) | 372743.7928 (539790.8095, 183158.8397) | 147.7677 (213.4399, 73.7502) |
| Republic of Paraguay | 190.8860 (275.1446, 102.1053) | 494.5706 (725.3961, 262.4518) | 8.8485 (12.9382, 4.8063) | 5017.4853 (7294.3458, 2221.1847) | 12142.4853 (18488.5281, 5793.9263) | 201.4063 (303.4237, 101.1602) |
| People's Democratic Republic of Algeria | 4179.7258 (5898.3547, 2019.4147) | 5464.5197 (7803.4478, 3279.8622) | 21.9902 (31.8009, 13.1990) | 113682.4521 (163022.8728, 50840.7714) | 122388.5773 (174285.4816, 71575.2337) | 376.1569 (525.2181, 225.4521) |
| Kingdom of Bahrain | 22.0512 (34.7185, 9.8981) | 38.1333 (56.6502, 19.2604) | 7.0285 (10.5095, 4.0046) | 715.2441 (1150.0313, 290.1763) | 1219.5161 (1887.8804, 575.3327) | 127.9953 (186.6676, 69.0267) |
| Arab Republic of Egypt | 11511.6774 (15816.9539, 7139.7165) | 8335.9122 (11827.8904, 5387.9186) | 19.1150 (26.9165, 12.3281) | 319446.8672 (442583.6755, 180040.6718) | 235933.7629 (338763.5508, 146768.3583) | 379.3828 (528.2372, 250.5003) |
| Islamic Republic of Iran | 2907.1181 (3924.7481, 1870.1192) | 4788.7763 (6544.7420, 3373.3722) | 7.0979 (9.7386, 5.0170) | 78228.9200 (105850.3628, 45176.2312) | 106743.3102 (143790.5335, 72220.3499) | 135.9535 (180.8978, 95.2415) |
| Republic of Iraq | 1831.3024 (2691.2958, 840.3011) | 5150.3925 (8135.0690, 2182.9325) | 24.9007 (37.9772, 11.6538) | 50292.7103 (75747.2335, 19360.2204) | 143910.6734 (235099.0946, 56375.6742) | 556.0257 (879.1603, 233.4016) |
| Hashemite Kingdom of Jordan | 345.3153 (477.9863, 206.0200) | 1061.7986 (1468.7295, 636.8039) | 18.6258 (25.2455, 12.0983) | 9724.4899 (13740.4703, 5338.6863) | 28269.8656 (39401.7131, 16037.6295) | 371.9845 (509.1941, 220.9063) |
| State of Kuwait | 100.2754 (133.4347, 61.7135) | 261.4403 (392.0072, 125.3275) | 9.0201 (13.3211, 4.7659) | 3315.2415 (4500.1746, 1818.4198) | 8441.4433 (13092.3410, 3745.7193) | 207.8847 (311.1920, 104.6812) |
| Lebanese Republic | 102.2975 (163.1048, 45.7509) | 235.2449 (347.2557, 131.0834) | 3.5372 (5.1509, 1.9457) | 2405.9849 (3731.1271, 1135.1372) | 4684.5692 (6860.4771, 2442.5532) | 73.5553 (108.3139, 38.0017) |
| State of Libya | 215.0144 (314.4102, 113.8541) | 782.5005 (1163.5924, 420.7282) | 16.4623 (23.8803, 9.0739) | 5995.6601 (8570.5927, 2956.7486) | 21960.5587 (33452.4535, 10801.1400) | 366.7704 (552.2079, 190.5951) |
| Kingdom of Morocco | 4623.8807 (6865.3879, 2154.4153) | 3521.6019 (5197.4199, 1930.0606) | 12.4352 (17.8312, 6.9508) | 126256.6506 (188226.4509, 54060.3935) | 82984.2078 (126075.4928, 44366.0018) | 248.8898 (367.1159, 138.0117) |
| Palestine | 354.1061 (508.9461, 185.6584) | 496.6134 (693.9233, 289.1599) | 27.1523 (37.0314, 17.2734) | 8035.2177 (11833.4191, 3837.8666) | 11984.7550 (17524.8199, 6457.3696) | 493.5240 (693.9308, 286.8764) |
| Sultanate of Oman | 140.6333 (204.3752, 84.5127) | 144.2987 (207.6047, 91.4340) | 9.8272 (14.0232, 6.2402) | 3881.0422 (5708.2481, 2092.3444) | 4369.9604 (6260.9610, 2642.3608) | 183.5293 (259.5867, 119.3822) |
| State of Qatar | 8.0998 (12.9177, 3.4576) | 15.0466 (27.2946, 6.1328) | 2.0435 (3.4654, 1.0259) | 309.6516 (517.8575, 116.1120) | 692.7228 (1265.2420, 235.3951) | 39.8394 (65.0140, 19.7240) |
| Kingdom of Saudi Arabia | 1348.0213 (1966.4679, 764.1060) | 4703.8309 (6948.9248, 2332.5090) | 25.6256 (36.4314, 14.1817) | 37377.1577 (56515.8020, 19797.9415) | 168786.2024 (255816.4553, 80556.3110) | 592.0496 (845.8395, 308.9123) |
| Syrian Arab Republic | 1126.7189 (1620.7171, 619.6160) | 2164.5646 (3409.3073, 1135.5831) | 21.5431 (32.4077, 12.2348) | 31423.5887 (46534.2947, 13316.8395) | 55660.9049 (90376.2636, 26087.2224) | 445.0237 (700.9174, 226.1698) |
| Republic of Tunisia | 1060.0566 (1484.9950, 635.5860) | 1817.2364 (2842.2265, 1007.6231) | 15.8331 (24.6743, 8.9460) | 25860.3163 (36586.4615, 14323.2541) | 37199.0214 (56767.3491, 20632.2772) | 294.8093 (451.0931, 164.6027) |
| Republic of Turkey | 3417.3404 (4943.1068, 1942.5605) | 3316.3172 (4938.6438, 1908.2348) | 4.1654 (6.2340, 2.3311) | 85898.7826 (125573.1145, 39445.1960) | 63026.4188 (89674.1563, 37898.0407) | 72.4734 (105.8011, 43.6224) |
| United Arab Emirates | 27.8659 (41.8118, 13.6650) | 239.3354 (355.4177, 124.6553) | 11.3305 (16.1650, 7.0189) | 1115.2978 (1654.6105, 491.0893) | 9562.7066 (14132.1034, 4732.8529) | 208.3572 (294.7780, 124.7424) |
| Republic of Yemen | 3523.4503 (5246.2545, 1558.2262) | 7565.8536 (11489.6894, 3601.2340) | 62.9593 (95.8551, 31.9514) | 101580.6300 (155399.6635, 40970.6995) | 209329.7941 (324905.5985, 90652.5550) | 1368.4182 (2081.3552, 642.8965) |
| Islamic Republic of Afghanistan | 6248.3427 (9059.5188, 2902.1073) | 6365.3650 (9570.6826, 3073.0082) | 71.7269 (106.0681, 38.4935) | 168772.2017 (250413.1506, 69635.3117) | 195837.9115 (300734.4214, 84269.7861) | 1651.8516 (2478.6144, 799.3029) |
| People's Republic of Bangladesh | 24463.2838 (37812.4626, 7819.2328) | 41030.6264 (64717.8761, 14978.1533) | 32.9876 (51.7531, 12.7202) | 731484.7510 (1147765.8565, 191643.7257) | 1083359.5054 (1760215.6124, 317394.7948) | 767.5006 (1234.1256, 248.6080) |
| Kingdom of Bhutan | 69.1900 (107.3386, 26.6422) | 119.9436 (184.2274, 53.2014) | 21.1606 (32.3466, 9.7699) | 2117.2459 (3315.6779, 720.0004) | 2903.8253 (4597.1314, 1122.8311) | 462.7684 (726.3403, 189.6459) |
| Republic of India | 177803.6736 (280907.6844, 55838.8106) | 413899.3531 (639180.0668, 132728.5579) | 36.5045 (56.3699, 12.5910) | 5629412.2444 (8798482.1379, 1627370.0362) | 11705334.2498 (18061315.5248, 3457897.6280) | 929.0292 (1428.6547, 287.1770) |
| Federal Democratic Republic of Nepal | 3128.8145 (4846.4509, 1163.7574) | 5552.5013 (8390.5138, 2637.2774) | 28.2063 (41.5868, 14.4856) | 93579.7991 (146703.2233, 29595.4074) | 138964.6988 (214950.4834, 57924.6020) | 602.0250 (919.9456, 268.9679) |
| Islamic Republic of Pakistan | 14979.7280 (23531.1996, 5699.1895) | 36637.9599 (59304.2765, 12916.4633) | 32.4432 (51.5449, 13.0686) | 422789.6927 (674642.8250, 139726.4681) | 1124995.4373 (1851702.9052, 354478.8454) | 793.6787 (1284.8114, 274.8606) |
| Republic of Angola | 1816.4043 (2609.5801, 942.1918) | 3124.5883 (4566.6644, 1624.3037) | 34.7069 (48.6418, 19.9505) | 53845.2357 (77611.1440, 26848.3489) | 88675.1722 (131044.7518, 42442.7445) | 728.4217 (1053.3887, 385.1150) |
| Central African Republic | 548.7439 (828.6233, 250.5368) | 954.5258 (1468.9960, 418.2070) | 53.1187 (79.7909, 26.1873) | 16332.4456 (24784.4995, 6989.9880) | 29470.9882 (45908.5162, 11804.8703) | 1214.5379 (1831.7471, 558.9915) |
| Republic of the Congo | 526.7330 (776.7271, 263.3549) | 891.0243 (1374.4465, 459.1393) | 40.2745 (60.0543, 22.1638) | 14982.8283 (22081.2115, 7185.9144) | 25805.0178 (39989.1109, 12349.4886) | 883.7028 (1350.1196, 462.5182) |
| Democratic Republic of the Congo | 4116.4336 (6232.4311, 1981.8264) | 12715.9838 (19017.5561, 5949.8942) | 44.3850 (63.4616, 22.6708) | 117906.1190 (180945.9156, 54285.4575) | 348481.5779 (527739.5846, 157421.8535) | 937.5277 (1388.8489, 445.4585) |
| Republic of Equatorial Guinea | 93.6530 (137.7105, 44.8110) | 76.6469 (128.4557, 38.3616) | 18.2206 (30.7395, 9.7765) | 2650.3345 (3983.5769, 1192.1438) | 2202.8017 (3712.2890, 1057.4123) | 388.7258 (642.4925, 194.4565) |
| Gabonese Republic | 76.7384 (114.5691, 46.6996) | 133.4630 (199.9160, 76.3793) | 15.9767 (24.5476, 9.1285) | 1888.7849 (2758.8216, 1069.3518) | 3474.8739 (5185.9052, 1983.6826) | 330.9442 (492.1930, 190.9860) |
| Republic of Burundi | 596.3507 (897.0640, 239.4560) | 622.4330 (919.0157, 279.4562) | 15.3706 (22.8394, 7.7693) | 17081.3443 (25775.7536, 6472.8682) | 18923.3622 (28319.6010, 8387.7079) | 341.2106 (501.1856, 157.2346) |
| Union of the Comoros | 44.8849 (65.6933, 24.0187) | 85.2626 (124.6315, 41.7999) | 20.4289 (30.4267, 10.5284) | 1274.7172 (1870.8508, 611.6294) | 2201.1301 (3187.6943, 1051.7425) | 439.2500 (639.1693, 214.5353) |
| Republic of Djibouti | 65.0630 (96.0804, 31.8243) | 212.6014 (348.9763, 91.8315) | 41.1440 (64.4044, 20.2628) | 2032.8015 (3069.0144, 962.5162) | 6562.3986 (10805.0346, 2676.0793) | 923.7421 (1493.5615, 417.9508) |
| State of Eritrea | 640.1416 (927.7954, 323.8060) | 1012.1317 (1512.3207, 529.5040) | 47.1985 (67.8018, 26.8603) | 20701.0612 (30344.8026, 9610.7633) | 30415.1321 (46870.1575, 14874.6143) | 1017.5533 (1497.2561, 543.7197) |
| Federal Democratic Republic of Ethiopia | 13133.5051 (18318.1184, 7159.5615) | 13517.5863 (18966.1196, 7188.1479) | 36.0623 (49.9024, 20.5242) | 382852.0893 (542063.3012, 192766.8779) | 357848.7977 (514712.7466, 170509.6827) | 783.2365 (1098.1265, 408.3798) |
| Republic of Kenya | 1588.0015 (2216.5309, 936.2319) | 4102.4813 (5848.7889, 2312.1442) | 22.8634 (31.6778, 13.8970) | 42295.2950 (59902.7602, 23060.0209) | 112918.1477 (164637.7428, 58172.5879) | 478.1889 (677.5035, 266.2609) |
| Republic of Madagascar | 2823.8495 (3935.6559, 1659.3467) | 5119.4896 (7439.4028, 2671.4333) | 53.8617 (77.0472, 30.8379) | 79908.2441 (112173.8190, 44228.9583) | 161839.4901 (240021.0258, 75648.3670) | 1242.2124 (1794.2579, 669.9859) |
| Republic of Malawi | 1036.1780 (1515.3608, 480.3455) | 1766.4703 (2690.5731, 856.0457) | 27.9792 (41.5883, 15.1215) | 29918.2604 (43872.3365, 12774.2431) | 51845.1746 (79967.3658, 21585.0091) | 632.2392 (958.7749, 307.5561) |
| Republic of Mauritius | 451.4143 (655.7413, 184.4270) | 423.1550 (592.0359, 217.5560) | 24.9140 (34.7506, 12.9804) | 13264.8374 (19313.0464, 4909.7895) | 10592.2853 (15087.3485, 4914.4989) | 615.2492 (875.3633, 285.6480) |
| Republic of Mozambique | 2584.3377 (3728.5756, 1276.8361) | 4642.9175 (6853.0611, 2028.7348) | 51.2063 (74.9879, 25.4914) | 70020.6818 (103884.5767, 29983.6562) | 134853.6621 (202605.9422, 48778.2292) | 1127.0519 (1654.5500, 499.6349) |
| Republic of Rwanda | 24.0197 (55.6117, 8.0269) | 64.6987 (127.4766, 25.5425) | 1.3575 (3.2734, 0.4189) | 845.4804 (1766.6104, 322.6710) | 2066.4218 (3725.8366, 944.2049) | 28.1925 (54.1953, 11.7046) |
| Republic of Seychelles | 26.8610 (34.3973, 17.8665) | 26.4917 (35.1651, 17.6598) | 25.6386 (33.7586, 17.1652) | 623.6613 (822.2539, 384.8592) | 642.1026 (859.7538, 415.3772) | 554.2750 (737.0226, 363.9914) |
| Federal Republic of Somalia | 1328.0471 (1968.0913, 634.8167) | 2495.7700 (3865.3051, 1068.2154) | 49.7075 (73.8189, 24.9988) | 41791.9056 (63626.3614, 17838.7056) | 79308.4182 (125113.9860, 31937.1825) | 1143.9142 (1726.5682, 508.0839) |
| United Republic of Tanzania | 3424.1080 (4779.1591, 1924.1091) | 4870.7867 (7513.2412, 2408.4712) | 23.4251 (35.5987, 12.3869) | 92267.7656 (131719.9545, 46716.6023) | 124631.4013 (190102.8781, 55240.4592) | 473.6491 (718.5080, 230.6369) |
| Republic of Uganda | 361.8548 (590.0204, 170.0709) | 910.7543 (1464.2990, 395.3890) | 7.0563 (11.6691, 3.2245) | 10645.2536 (17242.0143, 4567.1763) | 27919.9945 (43521.6173, 12233.9011) | 161.8573 (253.4786, 73.2676) |
| Republic of Zambia | 1766.4229 (2469.8155, 1027.3674) | 3644.2376 (5528.5865, 1781.2993) | 65.6544 (94.2064, 36.6783) | 48379.1537 (68178.5046, 25294.6380) | 102392.6029 (160242.0906, 45318.3554) | 1394.4150 (2100.9641, 706.9113) |
| Republic of Botswana | 255.6581 (399.7129, 119.5684) | 427.2146 (629.5420, 232.1363) | 35.5695 (50.3433, 21.0798) | 7183.9854 (11325.0759, 3163.1877) | 11537.4889 (17368.9820, 5688.9595) | 760.9722 (1119.4780, 406.5905) |
| Kingdom of Lesotho | 361.3046 (515.8907, 210.2772) | 636.9680 (927.8880, 324.9796) | 68.4266 (98.3379, 36.5774) | 8691.9375 (12473.9428, 4789.4185) | 17347.1896 (25688.2730, 8290.7173) | 1566.4206 (2281.1892, 785.6641) |
| Republic of Namibia | 296.9945 (433.9007, 158.7046) | 547.2680 (806.3349, 304.7392) | 48.4355 (69.1792, 27.8271) | 8085.3229 (11864.4858, 4033.9431) | 14230.2144 (21506.3006, 7169.2074) | 1024.0967 (1502.9189, 554.3504) |
| Republic of South Africa | 5564.3858 (7772.9839, 2989.4666) | 12492.6549 (16867.7236, 7450.2563) | 30.7796 (41.4239, 19.0841) | 168916.8790 (242074.0919, 82094.0963) | 329551.7676 (456814.1425, 179681.9961) | 687.9631 (942.3989, 395.0231) |
| Kingdom of Eswatini | 117.1582 (165.2868, 67.2484) | 194.6414 (294.5035, 106.8539) | 40.8065 (59.5557, 23.2238) | 3217.4599 (4541.1688, 1765.9586) | 5766.8667 (8854.9215, 2983.3917) | 944.2499 (1414.9414, 519.9185) |
| Republic of Zimbabwe | 1230.8258 (1791.6706, 576.1649) | 2949.9576 (4665.7076, 1224.3890) | 48.3472 (74.4740, 22.0656) | 32596.5543 (47338.8745, 14836.3477) | 88573.1565 (138697.5055, 35087.9138) | 1163.1269 (1824.1095, 485.7649) |
| Republic of Benin | 589.6641 (893.1752, 254.9250) | 1151.2058 (1716.0064, 490.8793) | 24.7839 (36.9133, 10.9248) | 16029.0679 (24229.6961, 6513.8198) | 32762.6547 (49170.2797, 13326.4467) | 574.8047 (860.5945, 244.9664) |
| Burkina Faso | 1682.6135 (2458.1285, 785.9507) | 3740.8832 (5430.5574, 1643.0039) | 45.1810 (65.5292, 21.5072) | 47416.5141 (69086.4616, 20563.7377) | 102039.1463 (147660.0059, 42939.7940) | 1040.2409 (1502.6136, 447.0972) |
| Republic of Cameroon | 793.1447 (1099.7094, 450.9478) | 1934.1863 (3079.4806, 928.0232) | 16.7656 (26.0260, 8.4940) | 22934.5170 (32147.0073, 12443.5116) | 60785.1530 (96596.9309, 28669.0063) | 406.3609 (641.9096, 193.2029) |
| Republic of Cabo Verde | 54.6633 (80.4705, 28.4420) | 89.3826 (137.7724, 39.0455) | 20.6821 (31.9785, 9.3762) | 1335.4974 (1977.3436, 638.6928) | 2164.1001 (3395.7374, 898.0108) | 462.5920 (721.7400, 193.0490) |
| Republic of Chad | 1091.3099 (1627.7524, 481.2094) | 2423.8956 (3812.5933, 1007.5049) | 45.5525 (70.9121, 20.2572) | 29527.7868 (44437.8636, 11710.5842) | 72049.3424 (111284.5160, 28861.0063) | 1114.6560 (1735.0336, 464.1770) |
| Republic of C么te d'Ivoire | 732.1853 (1075.7071, 320.0097) | 2112.7180 (3262.8230, 1028.3344) | 20.5699 (31.2374, 10.5065) | 23805.3622 (35067.5450, 9878.8786) | 66962.7558 (103393.4806, 31409.9020) | 497.5307 (767.2692, 243.0314) |
| Republic of the Gambia | 154.3440 (239.2226, 60.1591) | 489.9286 (752.0409, 189.0258) | 53.7384 (82.6346, 21.2961) | 4691.8422 (7249.5296, 1748.4160) | 14130.4046 (21987.5746, 5304.4255) | 1300.2197 (2003.1881, 499.1463) |
| Republic of Ghana | 1569.9016 (2281.0630, 717.4149) | 1642.3479 (2501.6605, 730.1604) | 10.5376 (15.7438, 5.0536) | 50311.0204 (73922.5554, 21982.1145) | 54255.4563 (81128.2136, 23107.9143) | 266.1502 (401.8916, 119.5513) |
| Republic of Guinea | 556.8486 (826.8244, 296.9653) | 1076.8384 (1619.2961, 559.1965) | 20.6131 (30.5310, 11.4047) | 15236.2711 (22806.3110, 7629.4915) | 30691.4720 (47673.3864, 14592.6152) | 491.0216 (747.4112, 246.0452) |
| Republic of Guinea-Bissau | 177.0912 (264.9100, 70.9690) | 285.1707 (432.4237, 115.4406) | 42.1529 (62.6714, 19.0057) | 5513.4838 (8300.3816, 2073.3819) | 9384.7573 (14461.6708, 3662.3628) | 1051.7767 (1586.2800, 433.0822) |
| Republic of Liberia | 308.5115 (447.2529, 147.4038) | 632.6309 (970.6262, 294.7278) | 31.9316 (47.6918, 15.4221) | 8499.1446 (12432.9024, 3877.0096) | 19602.0745 (30521.9134, 8854.1023) | 763.6341 (1173.1282, 359.0180) |
| Republic of Mali | 1188.3365 (1749.2008, 580.1675) | 1950.8683 (2940.8995, 869.4483) | 23.8820 (35.5271, 10.8579) | 35845.1132 (52694.8977, 16821.6950) | 59212.1888 (89349.2677, 26495.5885) | 593.2112 (883.3116, 270.5785) |
| Islamic Republic of Mauritania | 536.7983 (818.9486, 234.9713) | 797.6659 (1243.0796, 352.0794) | 40.4063 (62.9220, 18.3396) | 14731.0382 (22229.0099, 6015.1178) | 21281.0184 (33201.0569, 9737.6859) | 937.4321 (1465.9018, 428.8961) |
| Republic of the Niger | 956.8318 (1461.3234, 321.2980) | 2123.5163 (3353.9085, 800.5619) | 29.4304 (46.2773, 11.7004) | 29367.7407 (44484.9564, 9615.5617) | 62108.4479 (98342.3772, 22564.1516) | 698.2758 (1096.4544, 264.3861) |
| Federal Republic of Nigeria | 11678.3789 (17393.6242, 6319.1875) | 14420.5454 (21588.4222, 6758.9095) | 17.9696 (25.9604, 8.9967) | 307917.9788 (463433.5182, 156296.6521) | 412009.9779 (615028.8014, 186143.5620) | 407.7615 (608.9805, 189.5076) |
| Democratic Republic of Sao Tome and Principe | 8.5367 (12.6702, 3.7206) | 8.0812 (12.9866, 3.3287) | 7.8314 (12.6680, 3.6610) | 232.0133 (347.5356, 92.3437) | 254.3921 (411.8019, 98.0415) | 193.1710 (309.9136, 78.1048) |
| Republic of Senegal | 1193.2749 (1852.1526, 454.6419) | 2350.0900 (3794.0957, 1002.3769) | 32.9631 (52.5870, 14.3949) | 34905.1236 (53912.2642, 12631.3252) | 66336.0050 (106363.8148, 27622.1123) | 788.8382 (1273.8349, 333.3291) |
| Republic of Sierra Leone | 577.1166 (906.5561, 214.4752) | 1043.5076 (1638.7252, 335.2991) | 29.0403 (45.6611, 9.8047) | 16299.6287 (25329.4293, 5760.4221) | 31428.6863 (49414.4525, 9903.0106) | 720.9874 (1127.1305, 231.7434) |
| Togolese Republic | 528.6351 (830.1012, 182.4942) | 1613.7913 (2548.3804, 626.7771) | 45.8451 (72.2732, 18.8649) | 16434.4007 (25636.6828, 5282.4452) | 50265.2542 (79501.9627, 19538.6895) | 1136.8115 (1784.4327, 453.2899) |
| American Samoa | 4.6835 (7.3858, 1.6938) | 8.8816 (14.2012, 3.0203) | 19.7007 (31.0932, 7.2184) | 156.7718 (250.3431, 51.5953) | 270.7769 (443.1702, 81.7048) | 538.0843 (870.2548, 169.7523) |
| Bermuda | 5.4571 (8.2475, 2.4557) | 10.4023 (14.9841, 6.0346) | 7.1577 (10.4215, 3.9536) | 127.9744 (193.6870, 54.4275) | 203.5798 (300.0369, 104.8439) | 158.9702 (236.5762, 78.0948) |
| Cook Islands | 7.9158 (10.1416, 5.7355) | 6.9022 (9.1671, 4.8103) | 28.1827 (37.7548, 19.7759) | 207.5150 (273.9605, 143.0141) | 155.6458 (210.1981, 105.2487) | 652.1477 (885.4053, 442.8379) |
| Greenland | 5.8366 (9.2888, 1.7967) | 4.0709 (6.4143, 1.6016) | 6.5330 (10.2188, 2.5722) | 198.2887 (314.9654, 60.5031) | 122.2159 (190.1331, 48.4732) | 175.3116 (268.6576, 70.3607) |
| Guam | 17.1266 (22.8838, 10.3323) | 18.8017 (29.3159, 8.7073) | 9.1778 (14.3051, 4.1824) | 501.2961 (679.4903, 285.4895) | 583.0201 (909.4298, 261.6287) | 301.2351 (471.1023, 132.7690) |
| Principality of Monaco | 2.2220 (3.8893, 0.9899) | 1.8181 (2.9435, 0.9402) | 1.5558 (2.4979, 0.7922) | 41.6784 (70.1479, 16.3108) | 28.2584 (44.5765, 13.7852) | 32.2572 (51.8965, 14.2998) |
| Republic of Nauru | 2.4775 (3.9406, 0.7417) | 3.1940 (5.2931, 1.0048) | 52.7481 (85.3824, 18.0140) | 90.9031 (142.8474, 25.9416) | 115.9124 (193.3736, 33.4553) | 1547.0672 (2554.0523, 482.8484) |
| Republic of Niue | 0.8574 (1.3821, 0.3517) | 0.5504 (0.8953, 0.1998) | 26.7601 (43.4385, 9.7003) | 21.0017 (33.6117, 7.5312) | 14.5159 (23.5843, 4.7366) | 704.1260 (1142.6395, 229.0261) |
| Northern Mariana Islands | 2.6275 (4.4243, 0.6419) | 5.4630 (9.0912, 1.4688) | 11.5388 (18.8613, 3.5667) | 105.2935 (179.9697, 22.7088) | 173.2957 (291.3885, 43.1598) | 308.5602 (507.8186, 81.2513) |
| Republic of Palau | 2.8888 (4.7628, 0.7957) | 4.4760 (7.8693, 1.1675) | 21.7850 (36.1695, 6.0658) | 94.8340 (154.7485, 23.7315) | 148.3516 (259.1337, 37.0141) | 628.0907 (1069.5639, 159.0672) |
| Puerto Rico | 507.6906 (759.0360, 238.1765) | 452.6588 (632.3508, 292.6333) | 5.2794 (7.3926, 3.3067) | 11936.3569 (17784.2071, 5472.1216) | 7957.1554 (10967.5337, 4949.2969) | 119.9544 (169.5635, 70.5192) |
| Saint Kitts and Nevis | 18.4153 (28.0608, 7.5006) | 15.5142 (22.5336, 8.1637) | 27.0427 (38.3743, 15.7414) | 427.3161 (648.4712, 154.4129) | 377.2158 (551.8027, 182.7178) | 555.0003 (804.6085, 285.4084) |
| Republic of San Marino | 2.0009 (3.0948, 0.9779) | 2.3926 (3.7802, 1.3158) | 2.2427 (3.5769, 1.1374) | 35.7125 (54.8045, 14.1158) | 35.8101 (55.9357, 18.5025) | 44.4980 (72.4820, 21.6936) |
| Tokelau | 0.6363 (0.9608, 0.2702) | 0.4526 (0.7108, 0.1696) | 31.0869 (48.7957, 11.6479) | 16.3443 (25.6330, 6.2464) | 11.5903 (18.4594, 3.9318) | 809.1861 (1283.6287, 273.1970) |
| Tuvalu | 4.3557 (6.5804, 1.7167) | 4.5237 (6.9489, 1.6284) | 46.8244 (71.7799, 17.3843) | 133.7908 (203.0971, 48.5239) | 136.1537 (210.8379, 45.2316) | 1261.9685 (1941.7482, 428.0094) |
| United States Virgin Islands | 11.5505 (16.2782, 6.4451) | 9.5319 (14.5951, 5.5000) | 6.0502 (8.9564, 3.5289) | 317.2706 (450.2500, 168.9300) | 200.1934 (299.1834, 113.5057) | 138.2139 (202.9783, 77.7657) |
| Republic of South Sudan | 849.2221 (1278.4367, 448.6303) | 987.6392 (1569.0613, 466.2345) | 29.9249 (45.7041, 15.9423) | 22140.0037 (33668.7402, 10578.1860) | 28957.3998 (46515.7201, 12252.6711) | 683.1877 (1076.2890, 326.8559) |
| Republic of Sudan | 4505.9956 (6638.3521, 2023.0526) | 5387.5816 (8538.6186, 2568.6485) | 29.8937 (46.9866, 15.4730) | 130560.3081 (195887.8228, 53200.1543) | 157933.9368 (253830.6953, 66943.8419) | 691.3438 (1099.7270, 319.7919) |
